# Morita–Baylis–Hillman reaction of 3-formyl-9*H*-pyrido[3,4-*b*]indoles and fluorescence studies of the products

**DOI:** 10.3762/bjoc.18.92

**Published:** 2022-07-26

**Authors:** Nisha Devi, Virender Singh

**Affiliations:** 1 Department of Chemistry, DAV University, Jalandhar-Pathankot National Highway (NH 44), Jalandhar, 144012, Punjab, India; 2 Department of Chemistry, Central University of Punjab, Bathinda, India

**Keywords:** β-carboline, DABCO, fluorescence, MBH reaction, Michael addition, structure–fluorescence activity relationship

## Abstract

β-Carboline is a privileged class of the alkaloid family and is associated with a broad spectrum of biological properties. 3-Formyl-9*H*-pyrido[3,4-*b*]indole is a such potent precursor belonging to this family which can be tailored for installing diversity at various positions of β-carboline to generate unique molecular hybrids of biological importance. The present work is a step towards this and assimilates the results related to the exploration of 3-formyl-9*H*-β-carbolines for the synthesis of β-carboline C-3 substituted MBH adducts followed by evaluation of their fluorescent characteristic. The effect of contact time, solvent system, concentration and substituents was also studied during investigation of fluorescence properties of these derivatives.

## Introduction

**Figure 1:**
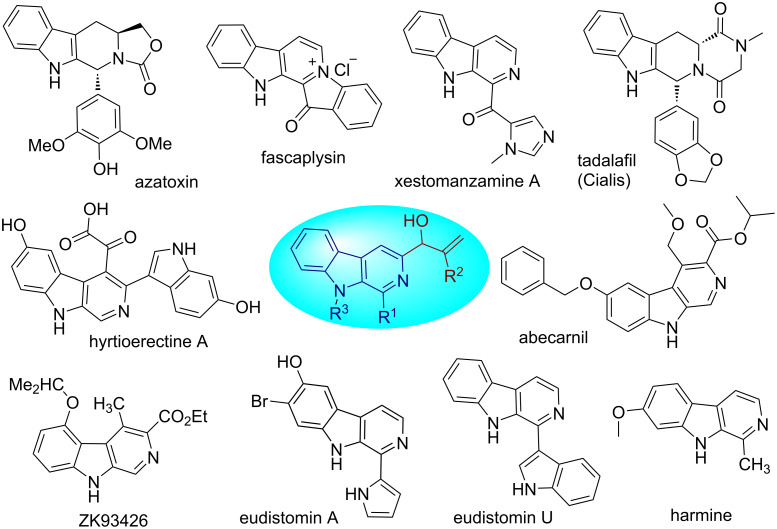
Few examples of β-carboline-based drugs and bioactive natural products.

Among the polycyclic alkaloids based on indole, the tricyclic structure β-carboline represents a promising class of pyridoindole alkaloids with a variety of biological activities which make them interesting synthetic targets [1–8]. Alkaloids containing the β-carboline nucleus in their molecular architecture are present ubiquitously in nature and a large number of natural products are reported representing this scaffold [9–16]. The key precursor used in the biosynthesis of β-carboline is ʟ-tryptophan which forms the basis of great abundance of β-carboline-containing natural products [17]. A broad spectrum of biological activities is displayed by this pharmacologically rich nucleus which includes antibacterial, antifungal, anticancer, anxiolytic, antimalarial, antiviral, anti-HIV, anti-Alzheimer, and anticonvulsant activities etc. [18–26]. Potent anticancer activities are shown by the majority of β-carboline-containing compounds [27–30]. Figure 1 summarizes some examples of β-carboline-based drugs and bioactive natural products some of which have even been commercialized successfully showing the importance of this nucleus [31–33]. This pharmacological richness and colossal medicinal importance is the reason that the synthesis of β-carboline-containing derivatives has been an exciting area for researchers [34–40].

The Morita–Baylis–Hillman (MBH) reaction is an astonishing C–C bond forming reaction between a carbonyl electrophile and an activated alkene leading to the formation of allylic alcohol; a highly functionalized product [41–44]. The chemistry of the MBH reaction is decorated with several unique features viz. atom economy, complexity generation and generation of a chiral center from a pro-chiral electrophile. The chemistry of the MBH reaction has gained considerable attention from the past two decades as these MBH adducts are highly functionalized and offer various points of diversity. Due to these amazing features, these MBH adducts act as starting material on which various organic transformations can be performed leading to the synthesis of various natural and synthetic products. MBH adducts itself display diverse biological activities like antifungal, antibacterial, herbicide, antiparasitic and antitumor as reviewed by Lima-Junior et al. (2012) [45].

**Figure 2:**
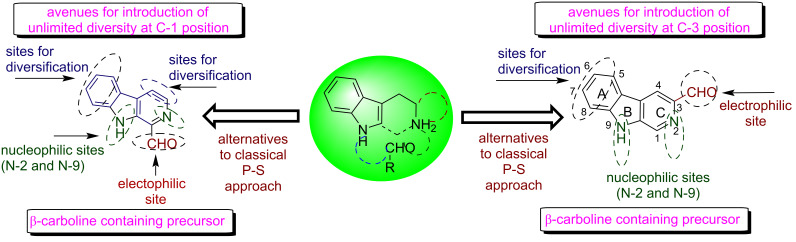
1/3-Formyl-9*H*-β-carboline: new synthons for the synthesis of β-carboline-fused and substituted frameworks.

It was envisaged that in comparison to the traditional methods like Pictet–Spengler (P-S) or Bischler–Napieralski (B-N) cyclisation, introduction of a formyl group at C-1 or C-3 position of the β-carboline frameworks may provide a new route for generating unlimited diversity at C-1 as well as at the C-3 position of β-carbolines. As depicted in Figure 2, 1-formyl-β-carbolines and 3-formyl-β-carbolines are decorated with different sites for diversification which make these synthons a promising template for the construction of β-carboline-fused frameworks via C-1 N-9, C-1 N-2 and C-3 N-2 cyclisation. Similarly, β-carboline-substituted molecular frameworks can be generated at C-3 position.

**Figure 3:**
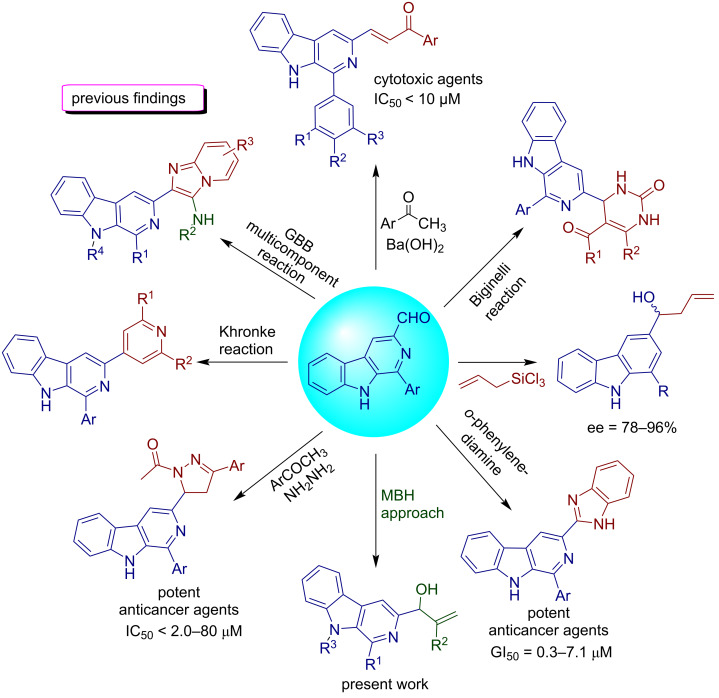
A summary of previous reports toward exploration of 3-formyl-9*H*-β-carbolines.

Our group has previously explored 1-formyl-β-carbolines and 3-formyl-β-carbolines for the generation of β-carboline-imidazo[1,2-*a*]azine conjugates at C-1 as well as C-3 position by the application of the Groebke–Blackburn–Bienaymé (GBB) multicomponent approach [46–47]. Our research group has also investigated the scope of 1-formyl-β-carbolines for generating unique molecular hybrids by application of the Morita–Baylis–Hillman reaction [48–50]. It was also revealed from a detailed literature survey that only limited reports have been documented toward exploration of 3-formyl-9*H*-β-carbolines for generating diversity at the β-carboline skeleton as outlined in Figure 3 [51–56].

Therefore, we herein report the synthesis of C-3-substituted pyrido[3,4-*b*]indole MBH adducts from substituted 3-formyl-9*H*-β-carbolines by the application of the MBH reaction followed by evaluation of their fluorescence properties.

## Results and Discussion

**Scheme 1:**
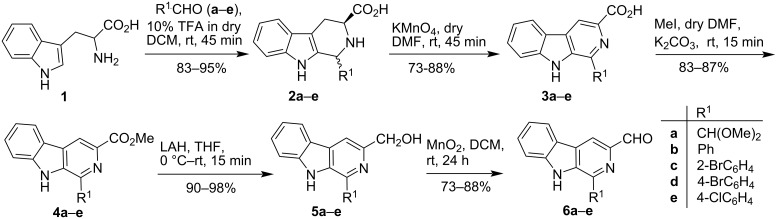
Synthesis of 3-formyl-9*H*-pyrido[3,4-*b*]indole derivatives.

The current study began with the synthesis of substituted 3-formyl-9*H*-β-carbolines (**6a**–**e**), which was accomplished by modifying the previously disclosed process as presented in Scheme 1 [46–47,57]. Pictet–Spengler (P-S) condensation of ʟ-tryptophan (**1**) with different aldehydes (**a**–**e**) in dry DCM at room temperature yielded tetrahydro-β-carboline derivatives **2a**–**e**, which were then oxidized with KMnO_4_ in anhydrous DMF for 45 minutes to yield β-carboline derivatives **3a**–**e**. It was encouraging to observe that the P-S condensation with ʟ-tryptophan (**1**) was much faster than with the tryptophan ester, taking only 45 minutes to complete. Interestingly, KMnO_4_ oxidation was selective, with no decarboxylation seen. Within 15 minutes, further treatment of **3a**–**e** with methyl iodide in the presence of K_2_CO_3_ provided the corresponding methyl ester **4a**–**e** in high yield (83–87%) and ester functionality reduction with LiAlH_4_ in dry THF yielded the alcohols **5a**–**e** in excellent yield (90–98%). The required 3-formyl-9*H*-β-carbolines **6a**–**e** were obtained in 73–88% yield by oxidizing the alcohol derivatives **5a**–**e** with MnO_2_ in dry DCM. The present methodology is decorated with several advantages like scalability and selectivity. Additionally, no column chromatographic purification was required at any stage and each step was high yielding.

**Scheme 2:**
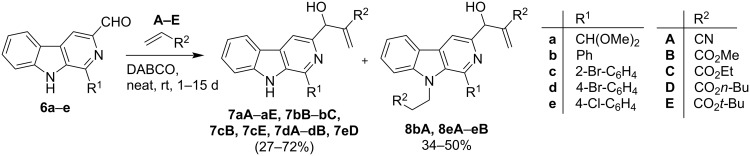
Synthesis of C-3 substituted pyrido[3,4-*b*]indole MBH derivatives (**7** and **8**).

After the synthesis of starting materials, the Morita–Baylis–Hillman reaction was explored for C-3 functionalization of the β-carboline framework. Accordingly, 3-formyl-9*H*-β-carbolines **6a**–**e** were subjected to MBH reaction with acrylonitrile **A** and various acrylates **B**–**E** under neat conditions in the presence of DABCO as depicted in Scheme 2. All the products were furnished smoothly in 27–72% yield. During this study, it was observed that the MBH reaction of **6b** with acrylonitrile **A** resulted in the formation of product **8bA** which evidenced that **6b** underwent Morita–Baylis–Hillman reaction at the electrophilic carbonyl center as well as Michael addition reaction at the nucleophilic nitrogen center (N-9). Similar results were obtained when **6e** was subjected to MBH reaction with acrylonitrile **A** and methylacrylate **B** and products **8eA** and **8eB** were generated as outlined in Scheme 2.

**Scheme 3:**
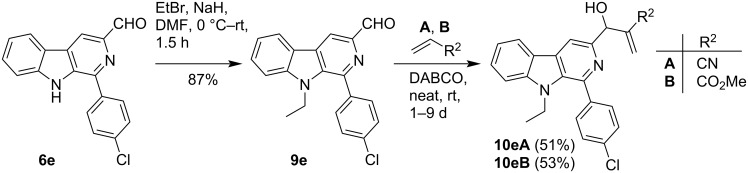
Synthesis of C-3 substituted pyrido[3,4-*b*]indole MBH derivatives **10**.

The effect of a substituent at N-9 position on the reactivity of 3-formyl-9*H*-pyrido[3,4-*b*]indole was also investigated during this study. For this purpose, the *N*-ethyl derivative **9e** of **6e** was prepared and subjected to MBH reaction with acrylonitrile **A** and methylacrylate **B** under neat conditions to generate the corresponding MBH adducts (**10eA** and **10eB**) (Scheme 3). Interestingly, **9e** showed more affinity towards this C–C bond forming transformation than **6e**. It is noteworthy here that all the products were purified by column chromatography.

**Figure 4:**
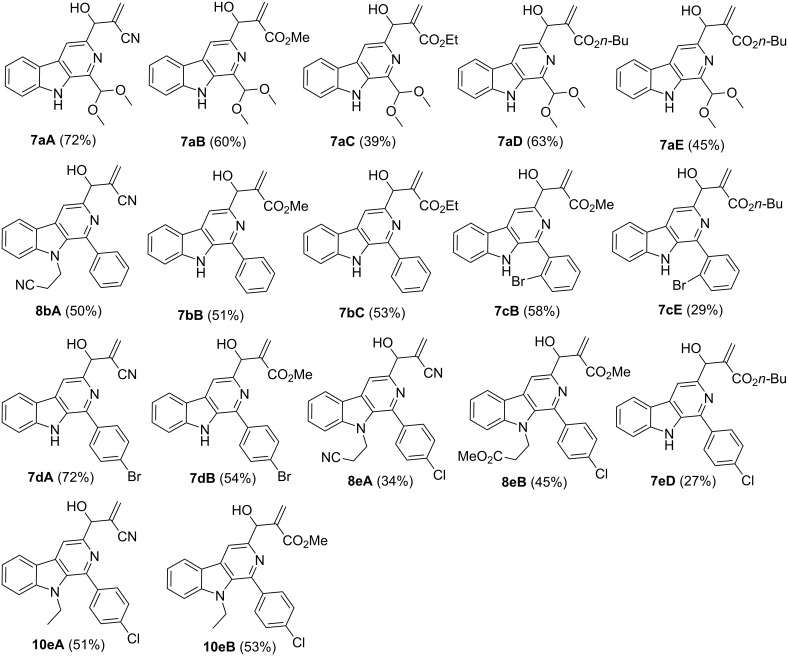
Library of C-3-substituted pyrido[3,4-*b*]indole MBH derivatives **7**, **8** and **10**.

A small library of C-3-substituted pyrido[3,4-*b*]indole derivatives was designed and synthesized which is presented in Figure 4. All the products were characterized using NMR, FTIR and mass spectrometry.

### Fluorescence studies

**Figure 5:**
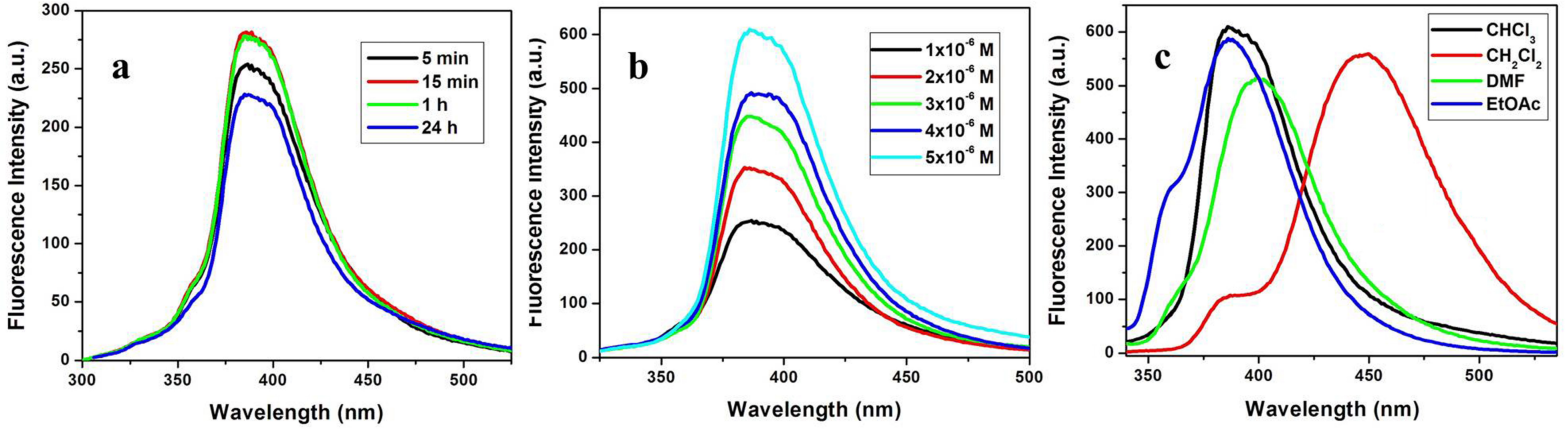
Results of optimization for fluorescence studies: a) contact time; b) concentration; c) solvent.

Fluorescence studies of these C-3-substituted pyrido[3,4-*b*]indole derivatives were examined and various parameters (contact time, concentration and solvent) were optimized for obtaining the best results using **7dA** as a model substrate. Fluorescence emission spectra for optimizing the contact time were recorded in chloroform at different intervals of time (5 min, 15 min, 1 h and 24 h) at 1 × 10^−6^ M concentration. **7dA** displayed the highest fluorescence intensity after 15 minutes and its fluorescence activity lasted even after 24 h with a slight decrease in fluorescence intensity. Further, the fluorescence emission profile of **7dA** was recorded in chloroform at different concentrations viz. 1 × 10^−6^ M, 2 × 10^−6^ M, 3 × 10^−6^ M, 4 × 10^−6^ M and 5 × 10^−6^ M which indicated that fluorescence intensity was found to increase with increase in concentration and fluorescence spectra above this concentration showed a fluorescence intensity >1000 a.u. After optimizing the time and concentration parameters, dilutions of **7dA** in different organic solvents such as dichloromethane, DMF and ethyl acetate were prepared for optimizing the solvent for obtaining the best fluorescence results. Fluorescence spectra were recorded after 15 minutes of sample preparation in 5 × 10^−6^ M concentration and fluorescence intensity was observed to be in the following order: CHCl_3_ > EtOAc > CH_2_Cl_2_ > DMF. The results of the optimization studies are presented in Figure 5 and it was concluded from the studies that C-3-substituted pyrido[3,4-*b*]indole derivative **7dA** displayed the maximum fluorescence intensity in chloroform at a concentration of 5 × 10^−6^ M after 15 minutes of sample preparation.

Accordingly, fluorescence studies of all the other derivatives were conducted following these optimized parameters, i.e., time: 15 min; concentration: 5 × 10^−6^ M; solvent: CHCl_3_. The results of the fluorescence studies of all the C-3 substituted pyrido[3,4-*b*]indole derivatives are presented in Table 1.

**Table 1:** Results of fluorescence studies of C-3-substituted pyrido[3,4-*b*]indole derivatives **7**–**8** and **10**.

sample	compound	R^1^	R^2^	λ_Ex_ (nm)	λ_Em_ (nm)	flourescence intensity (a.u.)

1	**7aA**	CH(OMe)_2_	CN	266	384	669.99
2	**7aB**	CH(OMe)_2_	CO_2_Me	260	369	624.79
3	**7aC**	CH(OMe)_2_	CO_2_Et	374	407	223.68
4	**7aD**	CH(OMe)_2_	CO_2_*n*-Bu	258	378	486.49
5	**7aE**	CH(OMe)_2_	CO_2_*t*-Bu	278	388	669.30
6	**8bA**	Ph	CN	278	391	592.48
7	**7bB**	Ph	CO_2_Me	278	384	768.75
8	**7bC**	Ph	CO_2_Et	278	383	766.89
9	**7cB**	2-Br-C_6_H_4_	CO_2_Me	278	375	>1000
10	**7cE**	2-Br-C_6_H_4_	CO_2_*t*-Bu	278	383	834.06
11	**7dA**	4-Br-C_6_H_4_	CN	278	386	609.70
12	**7dB**	4-Br-C_6_H_4_	CO_2_Me	294	386	768.78
13	**8eA**	4-Cl-C_6_H_4_	CN	270	395	385.38
14	**8eB**	4-Cl-C_6_H_4_	CO_2_Me	270	401	353.34
15	**7eD**	4-Cl-C_6_H_4_	CO_2_*n*-Bu	292	385	829.04
16	**10eA**	4-Cl-C_6_H_4_	CN	278	403	556.46
17	**10eB**	4-Cl-C_6_H_4_	CO_2_Me	270	428	415.32

### Structure–fluorescence activity relationships

**Figure 6:**
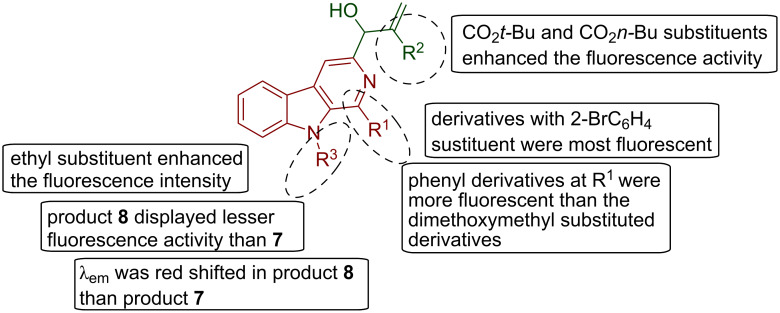
Pictorial representation of structure–fluorescence activity relationship of C-3 substituted pyrido[3,4-*b*]indole derivatives **7**, **8** and **10**.

From the results presented in Table 1, some structure–fluorescence activity relationships were concluded which are outlined in Figure 6. It was concluded from the structure–fluorescence activity relationships of C-3-substituted pyrido[3,4-*b*]indole derivatives that the products with *o*-bromophenyl substituent at R^1^ position (**7cB** and **7cE**) were the most fluorescent derivatives among all. Also, substituted phenyl derivatives were more fluorescent than the dimethoxymethyl substituted derivatives **7aA**–**7aE**. Further, it was observed that an ethyl substituent at N-9 position of β-carboline decreased the fluorescence intensity in **10eA**–**10eB** than **7eD** which is a *N*-unsubstituted derivative while the λ_emission_ was red shifted in *N*-ethyl-substituted derivatives as is clearly indicated from the data presented in Table 1. CO_2_*n*-Bu and CO_2_*t*-Bu substituents enhanced the fluorescence intensity more than the other substituents (**7aE**, **7cE** and **7eD**). It is noteworthy here that in the derivatives prepared from the same aldehydes, a noticeable decrease in fluorescence intensity of product **8** (Morita–Baylis–Hilman + Michael adducts **8bA**, **8eA** and **8eB**) was observed than in case of product **7** (Morita–Baylis–Hilman adducts **7bB**, **7bC** and **7eD**) while their λ_emission_ was red-shifted in comparison to type **7** compounds. This difference in the fluorescence intensity values of compound **7** and **8** may be attributed to the addition of a substituent at N-9 position of the β-carboline ring after Michael addition reaction (CH_2_CH_2_CN or CH_2_CH_2_CO_2_Me).

## Conclusion

In conclusion, we have successfully explored 3-formyl-1-aryl-9*H*-pyrido[3,4-*b*]indole derivatives for the C-3 functionalization by application of the MBH reaction to generate C-3-substituted β-carboline MBH adducts. It was revealed from spectroscopic analysis that few derivatives underwent MBH reaction as well as Michael addition reaction to form type **8** compounds. Additionally, the scope of the reaction was further extended and the effect of substituents at the N-9 position on the reactivity of 3-formyl-1-aryl-9*H*-pyrido[3,4-*b*]indoles was also investigated. Furthermore, fluorescence properties of these β-carboline conjugates were also studied and they were found to exhibit excellent fluorescence characteristics. Different parameters like contact time, concentration, solvent effects and substituent effects were examined for obtaining the optimal results. It was observed that the MBH derivatives exhibited excellent fluorescence characteristics at a concentration of 5 × 10^−6^ M in chloroform solvent after 15 minutes of sample preparation. Derivatives **7cB** and **7cE** bearing an *o*-bromophenyl substituent at R^1^ position emerged as two most fluorescent compounds in the present series. Furthermore, products of type **7** (Morita–Baylis–Hilman adducts) were more fluorescent than products **8** (Morita–Baylis–Hilman + Michael adducts). Antimicrobial evaluation of the title compounds is underway and will be reported in due course.

## Supporting Information

Supporting information contains detailed experimental procedure for the synthesis of compounds **6**–**9** and **10** followed by detailed characterization data and copies of ^1^H NMR and ^13^C NMR spectra of newly synthesized compounds **6**–**10**.

File 1 Experimental procedures and characterization data.
