# Supplementary material for: Morita–Baylis–Hillman reaction of 3-formyl-9H-pyrido[3,4-b]indoles and fluorescence studies of the products
Source: Beilstein J Org Chem. 2022 Jul 26;18:926–34. doi: 10.3762/bjoc.18.92 (PMC9344545; doi:10.3762/bjoc.18.92)
Supplement: File 1 — Experimental procedures and characterization data. [file Beilstein_J_Org_Chem-18-926-s001.pdf]

BEILSTEIN JOURNAL OF ORGANIC CHEMISTRY

Supporting Information

for

Morita–Baylis–Hillman reaction of 3-formyl-9*H*-pyrido[3,4-*b*]indoles and fluorescence studies of the products

Nisha Devi and Virender Singh

Beilstein J. Org. Chem. **2022,** *18,* 926–934. doi:10.3762/bjoc.18.92

Experimental procedures and characterization data

License and Terms: This is a supporting information file under the terms of the Creative Commons Attribution License (https://creativecommons.org/licenses/by/4.0). Please note that the reuse, redistribution and reproduction in particular requires that the author(s) and source are credited and that individual graphics may be subject to special legal provisions.
The license is subject to the *Beilstein Journal of Organic Chemistry* terms and conditions: (https://www.beilstein-journals.org/bjoc/terms)

Table of contents

Detailed experimental procedures, complete characterization data for new compounds **6–10**	S2–S11
^{1}H NMR and ^{13}C NMR spectra of new products **6–10**	S12–S30
References	S32

Experimental section

General section

The chemicals and reagents were purchased from Sigma-Aldrich, Acros, Avera Synthesis, Spectrochem Pvt. Ltd. and were used without further purification. Anhydrous solvents (CH_2Cl_2) utilised in the reactions were dried and freshly distilled before use. However, commercial anhydrous DMF and THF (Spectrochem make) were used as such without further distillation. Thin layer chromatography (TLC) was performed using pre-coated aluminium plates purchased from E. Merck (silica gel 60 PF254, 0.25 mm). Column chromatography was performed using Spectrochem silica gel (60–120 mesh). Melting points were determined in open head capillary tubes on a Precision Digital melting point apparatus (LABCO make) containing silicon oil and are uncorrected. The fluorescence spectra were recorded on Agilent Cary Eclipse spectrofluorimeter. The slit width for fluorescence experiments was at 5 nm (excitation) and 5 nm (emission) for all fluorescence studies. IR spectra were recorded using Agilent FTIR spectrophotometer. ^{1}H NMR and ^{13}C NMR spectra were recorded on Avance III Bruker spectrometer at operating frequencies of 400MHz (^{1}H) or 100 MHz (^{13}C) as indicated in the individual spectrum, using TMS as an internal standard. The MS spectra were recorded on Xevo G2-SQ TOF (Water, USA) or Thermo Finnigan LCQ Advantage, Ion Trap Mass Spectrometer. Elemental analyses were performed on a Carlo–Erba's 108 or an Elementar's Vario EL III microanalyzer. The room temperature varied between 25 °C and 35 °C. The multiplicity in ^{1}H NMR spectra is presented as s for singlet; d for doublet; dd for doublet of doublet; t for triplet, q for quartet and m for multiplet.

General procedure for the synthesis of compounds 2a–e as exemplified for 1-(4-bromophenyl)-2,3,4,9-tetrahydro-1*H*-pyrido[3,4-*b*]indole-3-carboxylic acid (2d): In a manner similar to the procedure described in [1], to a stirred suspension of L-tryptophan (**1**, 1.00 g, 4.89 mmol) in dry DCM (10 mL), 4- bromobenzaldehyde (**d**, 1.09 g, 5.89 mmol) was added at room temperature. Thereafter a solution of TFA (1 mL) in 5 mL dry DCM was added in small portions and the reaction was continued at room temperature till completion (45 min) which was monitored by TLC. On completion of the reaction, the excess of DCM was evaporated under reduced pressure and the crude product was washed with hexane 3–4 times (15 mL each time) to get the pure solid product **2d** as yellow solid (1.72 g from 1.00 g, 95%) which was significantly pure and utilized for the next step.

General procedure for the synthesis of compounds 3a–e as exemplified for 1-(4-bromophenyl)-9*H*-pyrido[3,4-*b*]indole-3-carboxylic acid (3d): To a stirred solution of tetra-hydro-β-carboline acid (**2d**, 1.72 g, 4.65 mmol) in dry DMF (10 mL), powdered $KMnO_4$ (3.00 g, 18.98 mmol) was added in small portions and stirred vigorously at room temperature for 45 min. After completion of the reaction as monitored by TLC, the blackish content was filtered through a bed of celite under suction to obtain a yellow filtrate. The residue over celite was further washed with a mixture of Methanol: DCM (05:95, v/v). The filtrate was concentrated in vacuo to yield the yellow solid product which was triturated with (hexane/EtOAc, 90:10, v/v) to obtain a light yellow solid product **3d** (1.49 g from 1.72 g, 88%).

General procedure for the synthesis of compounds 4a–e as exemplified for methyl 1-(4-bromophenyl)-9*H*-pyrido[3,4-*b*]indole-3-carboxylate (4d): To a stirred solution of β-carboline-3 carboxylic acid (**3d**, 1.49 g, 4.07 mmol) in dry DMF (10 mL), K_2CO_3 (1.40 g, 10.13 mmol) was added in small portions and stirred the reaction at room temperature for 15 min. Thereafter, methyl iodide (0.38 mL, 6.10 mmol) dissolved in DMF (1.5 mL) was added dropwise to the reaction mixture and the content was stirred at room temperature. After the completion of reaction as monitored by TLC, ice cold water was poured in the reaction mixture and compound was extracted with $CHCl_3$ (4 × 25 mL). The organic layers were combined and washed with brine (20 mL), dried over anhydrous Na_2SO_4, concentrated in vacuo to yield the solid product which was triturated with (hexane/EtOAc, 90:10, v/v) to obtain a yellow solid product **4d** (1.29 g from 1.49 g, 83%) which was sufficiently pure and utilized for next step without further purification.

General procedure for the synthesis of compounds 5a-e as exemplified for (1-(4-bromophenyl)-9*H*-pyrido[3,4-*b*]indol-3-yl)methanol (5d): To a stirred solution of β-carboline C-3 methyl ester (**4d**, 6.00 g, 15.75 mmol) in dry THF (70 mL), powdered LAH (1.49 g, 39.26 mmol) was added in small portions under nitrogen atmosphere and stirred the reaction at 0 °C. After 5 min of addition of LAH, the reaction was stirred room temperature till completion (10–15 min). The reaction mixture was quenched by pouring a saturated solution of NaOH drop wise into the reaction mixture under cooling conditions with constant stirring. The light grey reaction content was filtered through a bed of celite and further washed with a mixture of Methanol: DCM (05:95, v/v). The filtrate was concentrated in vacuo to yield the crude product which was triturated with (hexane/EtOAc, 90:10, v/v) to obtain a significantly pure yellow solid product, **5d** (5.45 g from 6.00 g, 98%).

General procedure for the synthesis of compounds 6a–e as exemplified for 1-(4-bromophenyl)-9*H*-pyrido[3,4-*b*]indole-3-carbaldehyde (6d): To a stirred solution of β-carboline alcohol (**5d**, 5.45 g, 15.51 mmol) in dry DCM (60 mL), powdered MnO_2 (20 g, 230 mmol) was added in small portions and stirred vigorously at room temperature. After the completion of reaction which was monitored by TLC, the blackish contents were filtered through a bed of celite under suction to obtain a yellow filtrate. The leftover residue over celite was further washed with dichloromethane. The filtrate was concentrated in vacuo to yield the crude solid product which was triturated with (hexane/EtOAc, 90:10, v/v) to obtain analytically pure yellow solid product **6d** (3.95 g from 5.46 g, 73%).

1-(Dimethoxymethyl)-9*H*-pyrido[3,4-*b*]indole-3-carbaldehyde (6a). Yield: 88% (3.96 g from 4.53 g) as yellow solid; m.p. 116-117 °C; R_f = 0.69 (hexane/EtOAc, 70/30, v/v); IR (neat): $\nu_{max}(cm^{-1})$ = 1716

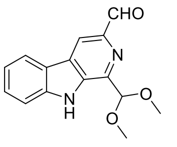

(CHO); ^{1}H NMR (400 MHz, $CDCl_3$) δ = 3.56 (s, 6 H, CH(OC*H*$_3$)$_2$), 5.78 (s, 1 H, C*H*(OCH$_3$)$_2$), 7.34-7.39 (m, 1 H, ArH), 7.58-7.62 (m, 2 H, ArH), 8.19 (d, *J* = 7.9 Hz, 1 H, ArH), 8.71 (s, 1 H, ArH), 9.47 (s, 1 H, $NH_{β\text{-carboline}}$) ppm, 10.23 (s, 1 H, CHO); ^{13}C NMR (100 MHz, $CDCl_3$) δ = 54.6, 106.4, 112.2, 114.8, 121.1, 121.4, 121.8, 129.3, 130.1, 135.8, 140.6, 140.7, 143.2, 193.2 ppm; MS (ES):*m/z* (%) = 271.1 $[M+1]^+$; $C_{15}H_{14}N_2O_3$ (270.1004): calcd. for C, 66.66; H, 5.22; N, 10.36; found C, 66.84; H, 5.25; N, 10.43.

General procedure for synthesis of C-3 linked β-carboline Morita–Baylis–Hillman adducts 7–8 and 10 as exemplified for 7aA: In a manner similar to [2], a solution of acrylonitrile (5 mL) and DABCO (0.31 g, 2.78 mmol) was stired at room temperature for 15 min. Thereafter, **6a** (0.50 g, 1.85 mmol) was added to the reaction mixture and stirring was continued at room temperature for additional 24 h. After completion of reaction as monitored by TLC, the contents were poured into water, extracted with EtOAc (3 x 10 mL) and washed with 10% $NaHCO_3$ solution (10 mL) followed by brine (10 mL). The organic layers were combined, dried over anhydrous Na_2SO_4 and concentrated *in vacuo.* The product was purified *via* silica gel (60-120 mesh) (hexane/EtOAc, 40/60, v/v) column chromatography to obtain the desired product **7aA** as off white solid (0.43 g from 0.50 g, 72%).

2-((1-(Dimethoxymethyl)-9*H*-pyrido[3,4-*b*]indol-3-yl)(hydroxy)methyl)acrylonitrile (7aA). Yield: 72% (0.43 g from 0.50 g) as off white solid; m.p. 89-90 °C; R_f = 0.53 (hexane/EtOAc, 50/50, v/v); IR

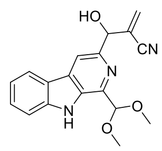

(neat): $\nu_{max}(cm^{-1})$ = 2254 (CN), 3226 (NH), 3339 (OH); ^{1}H NMR (400 MHz, $CDCl_3$) δ = 3.52 (s, 6 H, CH(OC*H*$_3$)$_2$), 4.11 (s, 1 H, CHO*H*), 5.48 (s, 1 H, C*H*(OCH$_3$)$_2$), 5.72 (s, 1 H, C*H*OH), 6.08 (s, 1 H, =CH*H*), 6.26 (s, 1 H, =C*H*H), 7.30 (t, *J* = 7.4 Hz, 1 H, ArH),

7.52-7.60 (m, 2 H, ArH), 7.96 (s, 1 H, ArH), 8.13 (d, *J* = 7.9 Hz, 1 H, ArH), 9.21 (s, 1 H, $NH_{\beta\text{-carboline}}$) ppm; ^{13}C NMR (100 MHz, $CDCl_3$) δ = 54.4, 54.5, 106.3, 111.8, 112.3, 117.2, 120.3, 120.8, 122.0, 127.0, 129.3, 130.5, 131.8, 133.7, 138.9, 141.2, 144.5 ppm; MS (ES):*m*/*z* (%) = 324.1 $[M+1]^+$; $C_{18}H_{17}N_3O_3$ (323.1270): calcd. for C, 66.86; H, 5.30; N, 13.00; found C, 67.05; H, 5.34; N, 13.11.

Methyl 2-((1-(dimethoxymethyl)-9*H*-pyrido[3,4-*b*]indol-3-yl)(hydroxy)methyl)acrylate (7aB). Yield: 60% (0.52 g from 0.65 g) as off white solid; m.p. 132-133 °C; R_f = 0.50 (hexane/EtOAc, 50/50, v/v); IR

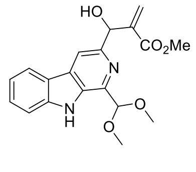

(neat): ν_{max}(cm^{-1}) = 1704 (CO_2Me), 3139 (NH), 3428 (OH); ^1H NMR (400 MHz, $CDCl_3$) δ = 3.49 (s, 3 H, CH(OC*H*3)), 3.52 (s, 3 H, CH(OC*H*3)), 3.74 (s, 3 H, CO_2CH_3), 4.90 (s, 1 H, CHO*H*), 5.70 (s, 1 H, C*H*$(OCH_3)_2$), 5.84 (s, 1 H, C*H*OH), 5.93 (s, 1 H, =CH*H*), 6.35 (s, 1 H, =C*H*H), 7.25-7.29 (m, 1 H, ArH), 7.50-7.57 (m, 2 H, ArH), 8.01 (s, 1 H, ArH), 8.12 (d, *J* = 7.9 Hz, 1 H, ArH), 9.12 (s, 1 H, $NH_{\beta\text{-carboline}}$) ppm; ^{13}C NMR (100 MHz, $CDCl_3$) δ = 51.9, 54.3, 54.5, 72.1, 106.7, 111.7, 112.3, 120.0, 121.1, 121.9, 126.3, 128.9, 131.4, 133.2, 138.6, 141.0, 142.9, 147.8, 167.0 ppm; MS (ES):*m*/*z* (%) = 357.1 $[M+1]^+$; $C_{19}H_{20}N_2O_5$ (356.1372): calcd. for C, 64.04; H, 5.66; N, 7.86; found C, 64.23; H, 5.71; N, 7.94.

Ethyl 2-((1-(dimethoxymethyl)-9*H*-pyrido[3,4-*b*]indol-3-yl)(hydroxy)methyl)acrylate (7aC). Yield: 39% (0.27 g from 0.50 g) as off white solid; m.p. 128-129 °C; R_f = 0.61 (hexane/EtOAc, 50/50, v/v); IR

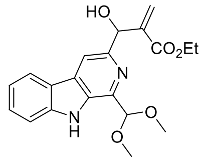

(neat): ν_{max}(cm^{-1}) = 1702 (CO_2Et), 3153 (NH), 3416 (OH); ^1H NMR (400 MHz, $CDCl_3$) δ = 1.23-1.27 (m, 3 H, CO_2CH_2C*H*3), 3.49 (s, 3 H, CH(OC*H*3)), 3.52 (s, 3 H, CH(OC*H*3)), 4.17-4.22 (m, 2 H, CO_2C*H*2CH_3), 4.90 (s, 1 H, CHO*H*), 5.69 (s, 1 H, C*H*$(OCH_3)_2$), 5.84 (s, 1 H, C*H*OH), 5.92 (s, 1 H, =CH*H*), 6.35 (s, 1 H, =C*H*H), 7.25 (t, *J* = 1.6 Hz, 1 H, ArH), 7.51-7.55 (m, 2 H, ArH), 8.02 (s, 1 H, ArH), 8.10 (d, *J* = 7.9 Hz, 1 H, ArH), 9.12 (s, 1 H, $NH_{\beta\text{-carboline}}$) ppm; ^{13}C NMR (100 MHz, $CDCl_3$) δ = 14.3, 54.4, 54.6, 60.9, 72.2, 106.8, 111.7, 112.4, 120.1, 121.2, 121.9, 126.2, 128.9, 131.4, 133.2, 138.6, 141.2, 143.1, 148.0, 166.6 ppm; MS (ES):*m*/*z* (%) = 371.1 $[M+1]^+$; $C_{20}H_{22}N_2O_5$ (370.1529): calcd. for C, 64.85; H, 5.99; N, 7.56; found C, 65.06; H, 6.04; N, 7.63.

Butyl 2-((1-(dimethoxymethyl)-9*H*-pyrido[3,4-*b*]indol-3-yl)(hydroxy)methyl)acrylate (7aD). Yield: 63% (0.23 g from 0.25 g) as off white solid; m.p. 89-90 °C; R_f = 0.65 (hexane/EtOAc, 50/50, v/v); IR (neat): ν_{max}(cm^{-1}) = 1706 ($CO_2{}^nBu$), 3207 (NH), 3416 (OH); ^1H NMR (400 MHz, $CDCl_3$) δ = 0.87 (t, *J* = 7.4 Hz, 3 H, $CO_2CH_2CH_2CH_2$C*H*3), 1.28-1.37 (m, 2 H, $CO_2CH_2CH_2$C*H*2CH_3), 1.56-1.63 (m, 2 H, CO_2CH_2C*H*2CH_2CH_3), 3.49 (s, 3 H, CH(OC*H*3)), 3.52 (s, 3 H, CH(OC*H*3)), 4.11-4.18 (m, 2 H,

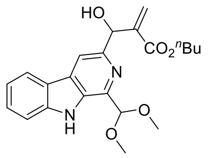

$CO_2CH_2CH_2CH_2CH_3$), 4.90 (d, *J* = 5.8 Hz, 1 H, CHO*H*), 5.69 (s, 1 H, C*H*($OCH_3)_2$), 5.84 (d, *J* = 4.9 Hz, 1 H, C*H*OH), 5.92 (s, 1 H, =CH*H*), 6.35 (s, 1 H, =C*H*H), 7.25 (t, *J* = 7.3 Hz, 1 H, ArH), 7.49-7.57 (m, 2 H, ArH), 8.02 (s, 1 H, ArH), 8.10 (d, *J* = 7.9 Hz, 1 H, ArH), 9.12 (s, 1 H, $NH_{β\text{-carboline}}$) ppm; ^{13}C NMR (100 MHz, $CDCl_3$) δ = 13.8, 19.3, 30.7, 54.3, 54.6, 64.8, 72.2, 106.7, 111.7, 112.3, 120.0, 121.1, 121.9, 126.2, 128.9, 131.4, 133.2, 138.6, 141.0, 143.1, 148.0, 166.7 ppm; MS (ES):*m/z* (%) = 399.1 $[M+1]^+$; $C_{22}H_{26}N_2O_5$ (398.1842): calcd. for C, 66.32; H, 6.58; N, 7.03; found C, 66.48; H, 6.64; N, 7.10.

***Tert*-butyl 2-((1-(dimethoxymethyl)-9*H*-pyrido[3,4-*b*]indol-3- yl)(hydroxy)methyl)acrylate (7aE).** Yield: 45% (0.35 g from 0.50 g) as off white solid; m.p. 147-148 °C; R_f = 0.61 (hexane/EtOAc, 50/50,

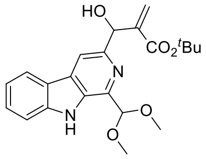

v/v); IR (neat): ν_{max}(cm^{-1}) = 1698 ($CO_2{}^tBu$), 3234 (NH), 3425 (OH); ^1H NMR (400 MHz, $CDCl_3$) δ = 1.44 (s, 9 H, $CO_2(CH_3)_3$), 3.48 (s, 3 H, CH(OC*H*3)), 3.53 (s, 3 H, CH(OC*H*3)), 4.84 (d, *J* = 5.9 Hz, 1 H, CHO*H*), 5.69 (s, 1 H, C*H*($OCH_3)_2$), 5.81 (s, 2 H, C*H*OH and =CH*H*), 6.26 (s, 1 H, =C*H*H), 7.24-7.28 (m, 1 H, ArH), 7.50-7.57 (m, 2 H, ArH), 8.03 (s, 1 H, ArH), 8.09 (d, *J* = 7.9 Hz, 1 H, ArH), 9.12 (s, 1 H, $NH_{β\text{-carboline}}$) ppm; ^{13}C NMR (100 MHz, $CDCl_3$) δ = 28.2, 54.3, 54.7, 72.4, 81.3, 106.8, 111.7, 112.2, 120.1, 121.2, 121.8, 125.4, 128.9, 131.4, 133.1, 138.6, 141.0, 144.4, 148.3, 166.0 ppm; MS (ES):*m/z* (%) = 399.1 $[M+1]^+$; $C_{22}H_{26}N_2O_5$ (398.1842): calcd. for C, 66.32; H, 6.58; N, 7.03; found C, 66.51; H, 6.66; N, 7.08.

2-((9-(2-Cyanoethyl)-1-phenyl-9*H*-pyrido[3,4-*b*]indol-3-yl)(hydroxy)methyl)acrylonitrile (8bA). Yield: 50% (0.35 from 0.50 g) as a light yellow solid; m.p. 144-145 °C; R_f = 0.70 (hexane/EtOAc, 50/50,

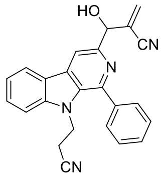

v/v); IR (neat): ν_{max}(cm^{-1}) = 2223 and 2264 (2 x CN), 3062 (OH); ^1H NMR (400 MHz, $CDCl_3$) δ = 2.30-2.33 (m, 2 H, CH_2CH_2CN), 4.34 (t, *J* = 7.4 Hz, 2 H, CH_2CH_2CN), 5.44 (s, 1 H, CHO*H*), 5.54 (s, 1 H, C*H*OH), 6.11 (s, 1 H, =CH*H*), 6.30 (s, 1 H, =C*H*H), 7.39 (t, *J* = 7.6 Hz, 1 H, ArH), 7.47 (d, *J* = 8.4 Hz, 1 H, ArH), 7.60-7.64 (m, 5 H, ArH), 7.65-7.69 (m, 1 H, ArH), 8.05 (s, 1 H, ArH), 8.21 (d, *J* = 7.9 Hz, 1 H, ArH) ppm; ^{13}C NMR (100 MHz, $CDCl_3$) δ = 17.2, 40.1, 73.2, 109.9, 111.2, 116.4, 117.2, 121.4, 121.7, 122.3, 126.6, 128.8, 129.3, 129.6, 129.7, 130.7, 132.8, 133.7, 138.6, 142.2, 142.7, 145.8 ppm; MS (ES):*m/z* (%) = 379.1 $[M+1]^+$; $C_{24}H_{18}N_4O$ (378.1481): calcd. for C, 76.17; H, 4.79; N, 14.81; found C, 76.38; H, 4.82; N, 14.88.

Methyl 2-(hydroxy(1-phenyl-9*H*-pyrido[3,4-*b*]indol-3-yl)methyl)acrylate (7bB). Yield: 51% (0.67 from 1.00 g) as white solid; m.p. 162-163 °C; R_f = 0.80 (hexane/EtOAc, 50/50, v/v); IR (neat): ν_{max}(cm^{-1}) = 1701 (CO_2Me), 3061 (NH), 3387 (OH); ^{1}H NMR (400 MHz, $CDCl_3$) δ = 3.77 (s, 3 H, CO_2CH_3), 5.19 (s, 1 H, CHO*H*), 5.90 (s, 1 H, C*H*OH), 6.02 (s, 1 H, =CH*H*), 6.37 (s, 1 H, =C*H*H), 7.30 (t, *J* = 7.4, 1 H, ArH), 7.48-7.55 (m, 3 H, ArH), 7.57-7.62 (m, 2 H, ArH), 7.98 (d, *J* = 7.8 Hz, 2 H, ArH), 8.01 (s, 1 H, ArH), 8.15 (d, *J* = 7.9 Hz, 1 H, ArH), 8.49 (s, 1 H, $NH_{\beta\text{-carboline}}$) ppm; ^{13}C NMR (100 MHz, $CDCl_3$) δ = 52.0, 72.0, 111.2, 111.7, 120.5, 122.0, 126.4, 128.3, 128.9, 129.1, 129.4, 133.1, 138.3, 141.1, 143.0, 149.2, 167.2 ppm; MS (ES):*m*/*z* (%) = 359.1 $[M+1]^+$; $C_{22}H_{18}N_2O_3$ (358.1317): calcd. for C, 73.73; H, 5.06; N, 7.82; found C, 73.89; H, 5.10; N, 7.88.

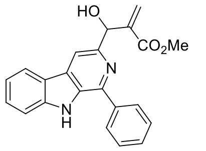

Ethyl 2-(hydroxy(1-phenyl-9*H*-pyrido[3,4-*b*]indol-3-yl)methyl)acrylate (7bC). Yield: 53% (0.36 from 0.50 g) as white solid; m.p. 138-139 °C; R_f = 0.86 (hexane/EtOAc, 50/50, v/v); IR (neat): ν_{max}(cm^{-1}) = 1699 (CO_2Et), 3094 (NH), 3339 (OH); ^{1}H NMR (400 MHz, $CDCl_3$) δ = 1.25 (t, *J* = 7.1 Hz, 3 H, $CO_2CH_2CH_3$), 4.17-4.26 (m, 2 H, $CO_2CH_2CH_3$), 5.22 (s, 1 H, CHO*H*), 5.90 (s, 1 H, C*H*OH), 6.01 (s, 1 H, =CH*H*), 6.38 (s, 1 H, =C*H*H), 7.30 (t, *J* = 7.4, 1 H, ArH), 7.50 (t, *J* = 7.8, 2 H, ArH), 7.54 (d, *J* = 7.0 Hz, 1 H, ArH), 7.60 (t, *J* = 7.5 Hz, 2 H, ArH), 7.97 (d, *J* = 8.0 Hz, 2 H, ArH), 8.02 (s, 1 H, ArH), 8.13 (d, *J* = 7.9 Hz, 1 H, ArH), 8.52 (s, 1 H, $NH_{\beta\text{-carboline}}$) ppm; ^{13}C NMR (100 MHz, $CDCl_3$) δ = 14.3, 60.9, 72.1, 111.2, 111.7, 120.5, 122.0, 122.1, 126.2, 128.2, 128.8, 129.1, 129.3, 131.3, 138.3, 141.1, 143.2, 149.2, 166.7 ppm; MS (ES):*m*/*z* (%) = 373.1 $[M+1]^+$; $C_{23}H_{20}N_2O_3$ (372.1474): calcd. for C, 74.18; H, 5.41; N, 7.52; found C, 74.39; H, 5.46; N, 7.58.

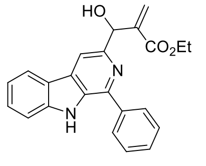

Methyl 2-((1-(2-bromophenyl)-9*H*-pyrido[3,4-*b*]indol-3-yl)(hydroxy)methyl)acrylate (7cB). Yield: 58% (0.36 from 0.50 g) as off white solid; m.p. 161-162 °C; R_f = 0.71 (hexane/EtOAc, 50/50, v/v); IR (neat): ν_{max}(cm^{-1}) = 1702 (CO_2Me), 3064 (NH), 3389 (OH); ^{1}H NMR (400 MHz, $CDCl_3$) δ = 3.77 (s, 3 H, CO_2CH_3), 5.16 (s, 1 H, CHO*H*), 5.90 (s, 1 H, C*H*OH), 6.02 (s, 1 H, =CH*H*), 6.37 (s, 1 H, =C*H*H), 7.30 (t, *J* = 7.5 Hz, 1 H, ArH), 7.48-7.56 (m, 2 H, ArH), 7.61 (t, *J* = 7.6 Hz, 2 H, ArH), 7.98 (d, *J* = 7.3 Hz, 2 H, ArH), 8.02 (s, 1 H, ArH), 8.15 (d, *J* = 7.9 Hz, 1 H, ArH), 8.47 (s, 1 H, $NH_{\beta\text{-carboline}}$) ppm; ^{13}C NMR (100 MHz, $CDCl_3$) δ = 52.0, 72.0, 111.2, 111.7, 120.5, 122.0, 122.2, 126.4, 128.3, 128.9, 129.1, 129.4, 131.3, 138.3, 141.1, 143.0, 149.2, 167.2 ppm; MS (ES):*m*/*z* (%) = 437.0 $[M+1]^+$, 439.0 $[M+3]^+$; $C_{22}H_{17}BrN_2O_3$ (436.0423): calcd. for C, 60.43; H, 3.92; N, 6.41; found C, 60.61; H, 3.95; N, 6.46.

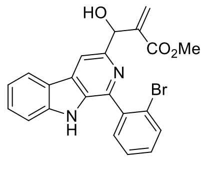

***tert*-Butyl 2-((1-(2-bromophenyl)-9*H*-pyrido[3,4-*b*]indol-3-yl)(hydroxy)methyl)acrylate (7cE).** Yield: 29% (0.20 from 0.50 g) as off white solid; m.p. 140-141 °C; R_f = 0.82 (hexane/EtOAc, 50/50, v/v);

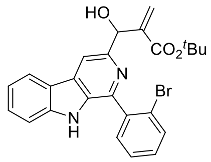

IR (neat): ν_{max}(cm^{-1}) = 1693 ($CO_2{}^tBu$), 3157 (NH), 3377 (OH); ^{1}H NMR (400 MHz, $CDCl_3$) δ = 1.43 (s, 9 H, $CO_2(CH_3)_3$), 5.17 (s, 1 H, CHO*H*), 5.86 (s, 1 H, C*H*OH), 5.92 (s, 1 H, =CH*H*), 6.28 (s, 1 H, =C*H*H), 7.30 (t, *J* = 7.9 Hz, 1 H, ArH), 7.51 (t, *J* = 6.9 Hz, 1 H, ArH), 7.55 (d, *J* = 7.0, 1 H, ArH), 7.60 (t, *J* = 7.5 Hz, 2 H, ArH), 7.98 (d, *J* = 8.3, 2 H, ArH), 8.02 (s, 1 H, ArH), 8.12 (d, *J* = 7.9 Hz, 1 H, ArH), 8.50 (s, 1 H, $NH_{\beta\text{-carboline}}$) ppm; ^{13}C NMR (100 MHz, $CDCl_3$) δ = 28.2, 72.3, 81.3, 111.0, 111.7, 120.5, 122.0, 125.4, 128.2, 128.8, 129.1, 129.3, 131.3, 133.0, 138.3, 140.9, 141.1, 144.5, 149.5 ppm; MS (ES):*m*/*z* (%) = 479.0 [M+1]$^+$, 481.0 [M+3]$^+$; $C_{25}H_{23}BrN_2O_3$ (478.0892): calcd. for C, 62.64; H, 4.84; N, 5.84; found C, 62.82; H, 4.89; N, 5.90.

2-((1-(4-Bromophenyl)-9*H*-pyrido[3,4-*b*]indol-3-yl)(hydroxy)methyl)acrylonitrile (7dA). Yield: 72%

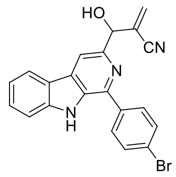

(0.34 from 0.30 g) as off white solid; m.p. 133-134 °C; R_f = 0.89 (hexane/EtOAc, 50/50, v/v); IR (neat): ν_{max}(cm^{-1}) = 2233 (CN), 3300 (NH and OH); ^{1}H NMR (400 MHz, $CDCl_3$) δ = 5.51 (s, 1 H, C*H*OH), 6.11 (s, 1 H, =CH*H*), 6.30 (s, 1 H, =C*H*H), 7.34 (t, *J* = 7.5 Hz, 1 H, ArH), 7.53 (d, *J* = 8.2 Hz, 1 H, ArH), 7.59 (t, *J* = 7.6 Hz, 1 H, ArH), 7.70 (d, *J* = 8.4 Hz, 2 H, ArH), 7.83 (d, *J* = 8.3 Hz, 2 H, ArH), 7.96 (s, 1 H, ArH), 8.15 (d, *J* = 7.9 Hz, 1 H, ArH), 8.56 (s, 1 H, $NH_{\beta\text{-carboline}}$) ppm; ^{13}C NMR (100 MHz, $CDCl_3$) δ = 73.4, 111.3, 111.9, 117.3, 120.9, 121.5, 122.1, 123.6, 127.0, 129.4, 129.7, 130.6, 131.9, 132.4, 133.4, 133.6, 136.5, 140.0, 141.3, 145.7 ppm; MS (ES):*m*/*z* (%) = 404.0 [M+1]$^+$, 406.0 [M+3]$^+$; $C_{21}H_{14}BrN_3O$ (403.0320): calcd. for C, 62.39; H, 3.49; N, 10.39; found C, 62.57; H, 3.53; N, 10.47.

Methyl 2-((1-(4-bromophenyl)-9*H*-pyrido[3,4-*b*]indol-3-yl)(hydroxy)methyl)acrylate (7dB). Yield:

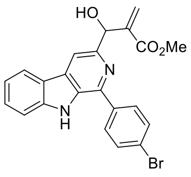

54% (0.33 from 0.50 g) as off white solid; m.p. 155-156 °C; R_f = 0.76 (hexane/EtOAc, 50/50, v/v); IR (neat): ν_{max}(cm^{-1}) = 1698 (CO_2Me), 3181 (NH), 3384 (OH); ^{1}H NMR (400 MHz, $CDCl_3$) δ = 3.76 (s, 3 H, CO_2CH_3), 5.13 (s, 1 H, CHO*H*), 5.89 (s, 1 H, C*H*OH), 6.04 (s, 1 H, =CH*H*), 6. 38 (s, 1 H, =C*H*H), 7.31 (t, *J* = 7.5 Hz, 1 H, ArH), 7.51 (d, *J* = 8.1 Hz, 1 H, ArH), 7.57 (t, *J* = 7.3 Hz, 1 H, ArH), 7.69 (d, *J* = 8.3 Hz, 2 H, ArH), 7.84 (d, *J* = 8.3 Hz, 2 H, ArH), 8.01 (s, 1 H, ArH), 8.13 (d, *J* = 7.9 Hz, 1 H, ArH), 8.57 (s, 1 H, $NH_{\beta\text{-carboline}}$) ppm; ^{13}C NMR (100 MHz, $CDCl_3$) δ = 52.0, 72.2, 111.5, 111.7, 120.6, 121.9, 122.1, 123.3, 126.5, 129.0, 129.8, 132.4, 132.9,

137.1, 141.2, 142.8, 149.2, 167.1 ppm; MS (ES):*m/z* (%) = 437.0 [M+1]$^+$, 439.0 [M+3]$^+$; $C_{22}H_{17}BrN_2O_3$ (436.0423): calcd. for C, 60.43; H, 3.92; N, 6.41; found C, 60.64; H, 3.95; N, 6.47.

2-((1-(4-Chlorophenyl)-9-(2-cyanoethyl)-9*H*-pyrido[3,4-*b*]indol-3-yl)(hydroxy)methyl) acrylonitrile (8eA). Yield: 34% (0.20 from 0.50 g) as off white solid; m.p. 138-139 °C; R_f = 0.73

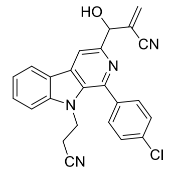

(hexane/EtOAc, 50/50, v/v); IR (neat): ν_{max}(cm^{-1}) = 2225 (CN) , 2255 (CN), 3323(OH); ^{1}H NMR (400 MHz, $CDCl_3$) δ = 2.31-2.36 (m, 2 H, CH_2CH_2CN), 4.39 (t, J = 7.1 Hz, 2 H, CH_2CH_2CN), 5.51 (s, 1 H, C*H*OH), 6.11 (s, 1 H, =CH*H*), 6.29 (s, 1 H, =C*H*H), 7.40 (t, J = 7.6 Hz, 1 H, ArH), 7.48 (d, J = 8.4 Hz, 1 H, ArH), 7.56-7.62 (m, 4 H, ArH), 7.65-7.70 (m, 1 H, ArH), 8.04 (s, 1 H, ArH), 8.21 (d, J = 7.8 Hz, 1 H, ArH) ppm; ^{13}C NMR (100 MHz, $CDCl_3$) δ = 17.2, 40.1, 73.2, 110.1, 111.4, 116.3, 117.2, 121.6, 121.8, 122.4, 126.6, 129.1, 129.9, 130.8, 133.2, 133.7, 135.8, 137.0, 141.4, 142.3, 146.1 ppm; MS (ES):*m/z* (%) = 413.1 [M+1]$^+$, 415.1 [M+3]$^+$; $C_{24}H_{17}ClN_4O$ (412.1091): calcd. for C, 69.82; H, 4.15; N, 13.57; found C, 69.99; H, 4.19; N, 13.64.

Methyl 2-((1-(4-chlorophenyl)-9-(3-methoxy-3-oxopropyl)-9*H*-pyrido[3,4-*b*]indol-3-yl)(hydroxy)-methyl)acrylate (8eB). Yield: 45% (0.35 from 0.50 g) as a light yellow solid; m.p. 133-134 °C; R_f = 0.68

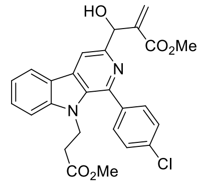

(hexane/EtOAc, 50/50, v/v); IR (neat): ν_{max}(cm^{-1}) = 1725 (2 x CO_2Me), 3171 (OH); ^{1}H NMR (400 MHz, $CDCl_3$) δ = 2.30 (t, J = 7.5 Hz, 2 H, $CH_2CH_2CO_2CH_3$), 3.47 (s, 3 H, $CH_2CH_2CO_2CH_3$), 3.77 (s, 3 H, CO_2CH_3), 4.34 (t, J = 7.5 Hz, 2 H, $CH_2CH_2CO_2CH_3$), 4.97 (s, 1 H, CHO*H*), 5.87 (s, 1 H, C*H*OH), 5.98 (s, 1 H, =CH*H*), 6.36 (s, 1 H, =C*H*H), 7.31 (t, J = 7.5 Hz, 1 H, ArH), 7.47 (d, J = 8.4 Hz, 1 H, ArH), 7.51 (d, J = 8.4 Hz, 2 H, ArH), 7.56 (d, J = 8.4 Hz, 2 H, ArH), 7.60 (t, J = 8.1 Hz, 1 H, ArH), 8.06 (s, 1 H, ArH), 8.16 (d, J = 7.8 Hz, 1 H, ArH) ppm; ^{13}C NMR (100 MHz, $CDCl_3$) δ = 33.4, 40.3, 52.0, 71.9, 110.3, 111.4, 120.6, 121.7, 122.0, 126.4, 128.7, 129.2, 130.9, 132.5, 135.0, 138.0, 141.1, 142.7, 148.6, 167.1, 171.0 ppm; MS (ES):*m/z* (%) = 479.1 [M+1]$^+$, 481.1 [M+3]$^+$; $C_{26}H_{23}ClN_2O_5$ (478.1295): calcd. for C, 65.20; H, 4.84; N, 5.85; found C, 65.41; H, 4.89; N, 5.92.

Butyl 2-((1-(4-chlorophenyl)-9*H*-pyrido[3,4-*b*]indol-3-yl)(hydroxy)methyl)acrylate (7eD). Yield:

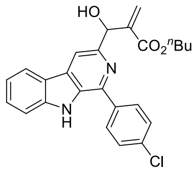

27% (0.19 from 0.50 g) as a light yellow solid; m.p. 137-138 °C; R_f = 0.90 (hexane/EtOAc, 50/50, v/v); IR (neat): ν_{max}(cm^{-1}) = 1708 ($CO_2{}^n$Bu), 3280 (NH), 3484 (OH); ^{1}H NMR (400 MHz, $CDCl_3$) δ = 0.84 (t, J = 7.4 Hz, 3 H, $CO_2CH_2CH_2CH_2CH_3$), 1.25-1.33 (m, 2 H, $CO_2CH_2CH_2CH_2CH_3$), 1.55-1.62 (m, 2 H, $CO_2CH_2CH_2CH_2CH_3$), 4.12-4.16 (m, 2 H,

$CO_2CH_2CH_2CH_2CH_3$), 5.12 (s, 1 H, CHO*H*), 5.88 (s, 1 H, C*H*OH), 6.02 (s, 1 H, =CH*H*), 6.39 (s, 1 H, =C*H*H), 7.31 (t, *J* = 7.4 Hz, 1 H, ArH), 7.50-7.61 (m, 4 H, ArH), 7.91 (d, *J* = 8.0 Hz, 2 H, ArH), 8.01 (s, 1 H, ArH), 8.12 (d, *J* = 7.9 Hz, 1 H, ArH), 8.53 (s, 1 H, $NH_{\beta\text{-carboline}}$) ppm; ^{13}C NMR (100 MHz, $CDCl_3$) δ = 13.8, 19.3, 30.7, 64.8, 72.4, 111.5, 111.7, 120.7, 121.9, 122.2, 126.3, 129.0, 129.5, 131.6, 133.0, 135.1, 136.7, 141.2, 143.0, 149.4, 166.7 ppm; MS (ES):*m/z* (%) = 435.1 $[M+1]^+$, 437.1 $[M+3]^+$; $C_{25}H_{23}ClN_2O_3$ (434.1397): calcd. for C, 69.04; H, 5.33; N, 6.44; found C, 69.24; H, 5.37; N, 6.49.

1-(4-Chlorophenyl)-9-ethyl-9*H*-pyrido[3,4-*b*]indole-3-carbaldehyde (9e) from 6e: To stirred solution of **6e** (3.00 g, 9.78 mmol) in dry DMF (15 mL), NaH (0.06 g, 2.42 mmol) was added at 0 °C and stirred the reaction content for 15 minutes. Thereafter ethyl bromide (0.096 µL, 0.89 mmol) was added drop wise to the reaction mixture at 0 °C and after 5 minutes, the reaction mixture was transferred to room temperature. After completion of reaction, as investigated by TLC, the contents were poured into ice cold water (20 mL) under stirring with glass rod which resulted in the formation of yellow solid. The solid product was filtered under vacuum and dried in air to yield a white solid **9e** (2.84 g from 3.00 g; 87%). The resulting product was analytically pure and we proceeded for next step without further purification.

1-(4-Chlorophenyl)-9-ethyl-9*H*-pyrido[3,4-*b*]indole-3-carbaldehyde (9e). Yield: 87% (2.84 g from 3.00 g) as yellow solid; m.p. 159-160 °C; R_f = 0.74 (hexane/EtOAc, 70/30, v/v); IR (neat): $\nu_{max}(cm^{-1})$ = 1712 (CHO); ^1H NMR (400 MHz, $CDCl_3$) δ = 0.99 (t, *J* = 7.0 Hz, 3 H, NCH_2CH_3), 3.75 (q, *J* = 7.0 Hz, 2 H, NCH_2CH_3), 7.33 (t, *J* = 7.5 Hz, 1 H, ArH), 7.46 (d, *J* = 8.3 Hz, 1 H, ArH), 7.51 (d, *J* = 8.2 Hz, 2 H, ArH), 7.57-7.64 (m, 3 H, ArH), 8.17 (s, 1 H, ArH), 8.22 (d, *J* = 8.0 Hz, 1 H, ArH), 10.31 (s, 1 H, CHO) ppm; ^{13}C NMR (100 MHz, $CDCl_3$) δ = 14.2, 39.6, 110.7, 113.6, 121.4, 122.1, 125.5, 129.4, 130.9, 131.1, 131.6, 131.8, 136.2, 138.4, 142.3, 142.9, 193.4 ppm; MS (ES):*m/z* (%) = 335.1 $[M+1]^+$, 337.1 $[M+3]^+$; $C_{20}H_{15}ClN_2O$ (334.0873): calcd. for C, 71.75; H, 4.52; N, 8.37; found C, 71.96; H, 4.56; N, 8.46.

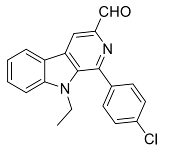

2-((1-(4-Chlorophenyl)-9-ethyl-9*H*-pyrido[3,4-*b*]indol-3-yl)(hydroxy)methyl)acrylonitrile (10eA). Yield: 51% (0.88 from 1.50 g) as white solid; m.p. 149-150 °C; R_f = 0.74 (hexane/EtOAc, 50/50, v/v); IR (neat): $\nu_{max}(cm^{-1})$ = 2249 (CN), 1732 (CO_2Et), 3089 (OH); ^1H NMR (400 MHz, $CDCl_3$) δ = 1.00 (t, *J* = 7.1 Hz, 3 H, NCH_2CH_3), 4.06 (q, *J* = 7.1 Hz, 2 H, NCH_2CH_3), 5.49 (s, 2 H, CHO*H* and C*H*OH), 6.09 (s, 1 H, =CH*H*), 6.28 (s, 1 H, =C*H*H), 7.34 (t, *J* = 7.5 Hz, 1 H, ArH), 7.47 (d, *J* = 8.4 Hz, 1 H, ArH), 7.52 (d, *J* = 8.2 Hz, 2 H, ArH), 7.59 (d, *J* = 8.2 Hz, 2

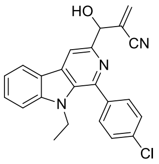

H, ArH), 7.63 (t, J = 7.8 Hz, 1 H, ArH), 8.02 (s, 1 H, ArH), 8.19 (d, J = 7.9 Hz, 1 H, ArH) ppm; ^{13}C NMR (100 MHz, $CDCl_3$) δ = 14.0, 39.5, 73.2, 110.4, 111.2, 117.3, 120.5, 121.4, 122.1, 126.9, 128.6, 129.3, 130.5, 130.8, 132.5, 134.2, 135.1, 137.9, 141.4, 142.9, 144.5 ppm; MS (ES):*m*/*z* (%) = 388.1 $[M+1]^+$, 390.1 $[M+3]^+$; $C_{23}H_{18}ClN_3O$ (387.1138): calcd. for C, 71.22; H, 4.68; N, 10.83; found C, 71.41; H, 4.71; N, 10.89.

Methyl 2-((1-(4-chlorophenyl)-9-ethyl-9*H*-pyrido[3,4-*b*]indol-3- yl)(hydroxy)methyl)acrylate

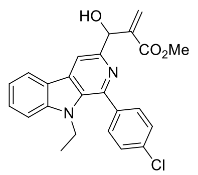

(10eB). Yield: 53% (0.99 from 1.50 g) as white solid; m.p. 154-155 °C; R_f = 0.77 (hexane/EtOAc, 50/50, v/v); IR (neat): ν_{max}(cm^{-1}) = 1702 (CO_2Me), 3109 (OH); ^{1}H NMR (400 MHz, $CDCl_3$) δ = 0.98 (t, J = 7.0 Hz, 3 H, NCH_2CH_3), 3.77 (s, 3 H, CO_2CH_3), 4.03 (q, J = 7.0 Hz, 2 H, NCH_2CH_3), 5.02 (s, 1 H, CHO*H*), 5.87 (s, 1 H, C*H*OH), 5.98 (s, 1 H, =CH*H*), 6.35 (s, 1 H, =C*H*H), 7.30 (t, J = 7.5 Hz, 1 H, ArH), 7.44 (d, J = 8.5 Hz, 1 H, ArH), 7.51 (d, J = 8.6 Hz, 2 H, ArH), 7.56-7.62 (m, 3 H, ArH), 8.05 (s, 1 H, ArH), 8.17 (d, J = 7.8 Hz, 1 H, ArH) ppm; ^{13}C NMR (100 MHz, $CDCl_3$) δ = 13.9, 39.4, 52.0, 71.8, 110.3, 111.3, 120.2, 121.7, 122.0, 126.3, 128.5, 128.9, 130.8, 132.2, 133.7, 134.8, 138.4, 141.1, 142.7, 142.8, 148.1, 167.1 ppm; MS (ES):*m*/*z* (%) = 421.1 $[M+1]^+$, 423.1 $[M+3]^+$; $C_{24}H_{21}ClN_2O_3$ (420.1241): calcd. for C, 68.49; H, 5.03; N, 6.66; found C, 68.66; H, 5.07; N, 6.7.

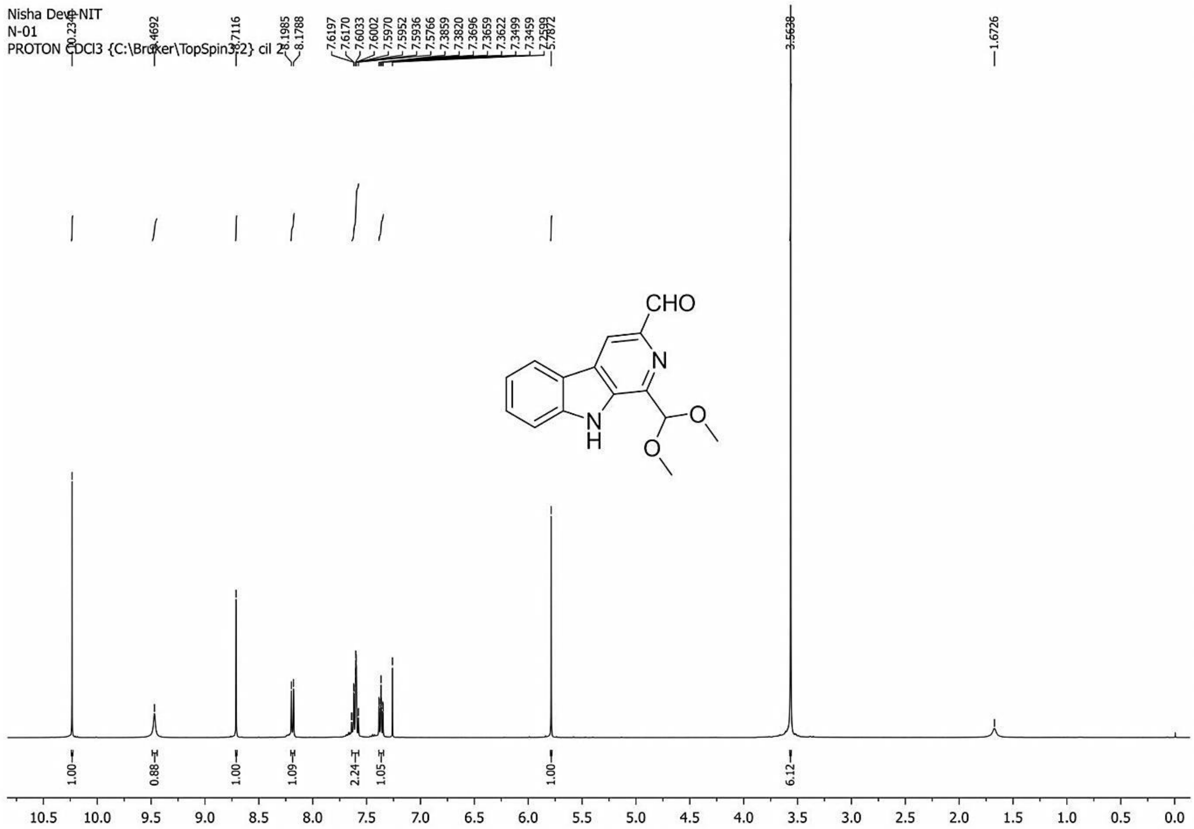

Fig. S1. ^{1}H NMR of 1-(dimethoxymethyl)-9*H*-pyrido[3,4-*b*]indole-3-carbaldehyde (**6a**).

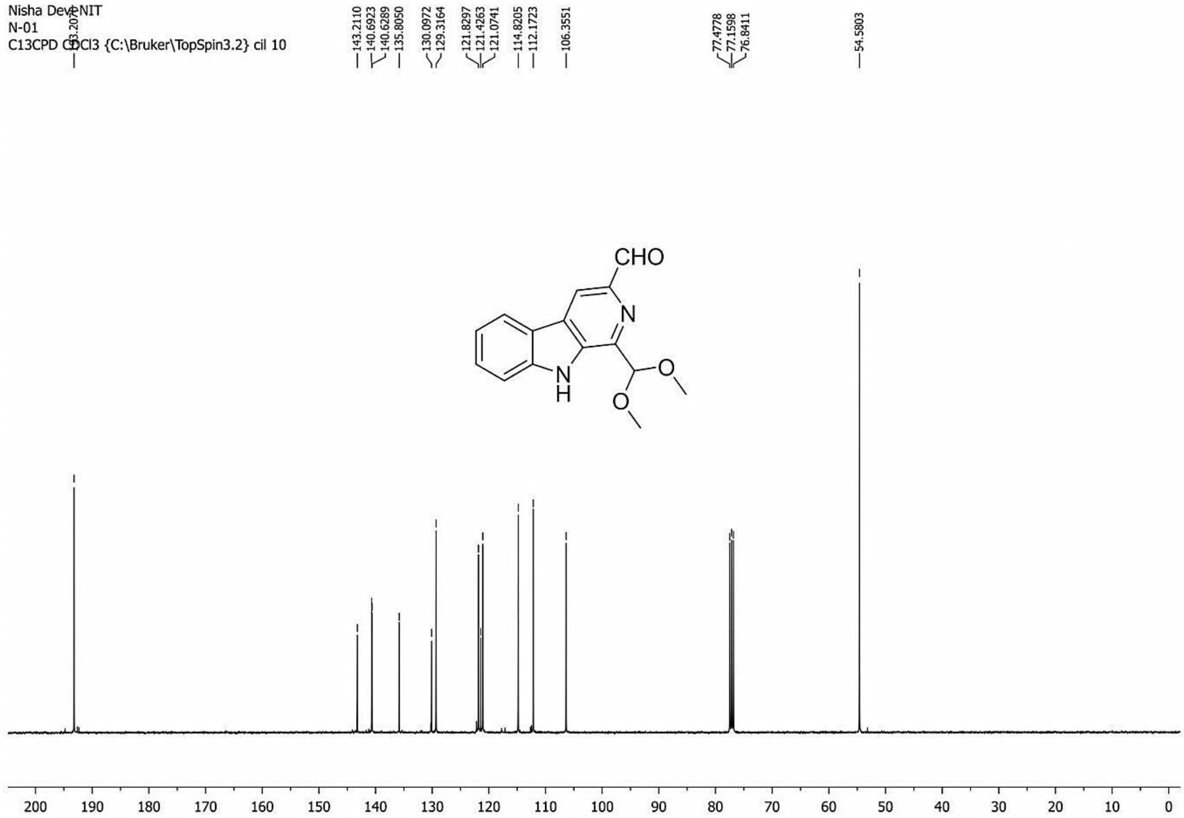

Fig. S2. ^{13}C NMR of 1-(dimethoxymethyl)-9*H*-pyrido[3,4-*b*]indole-3-carbaldehyde (**6a**).

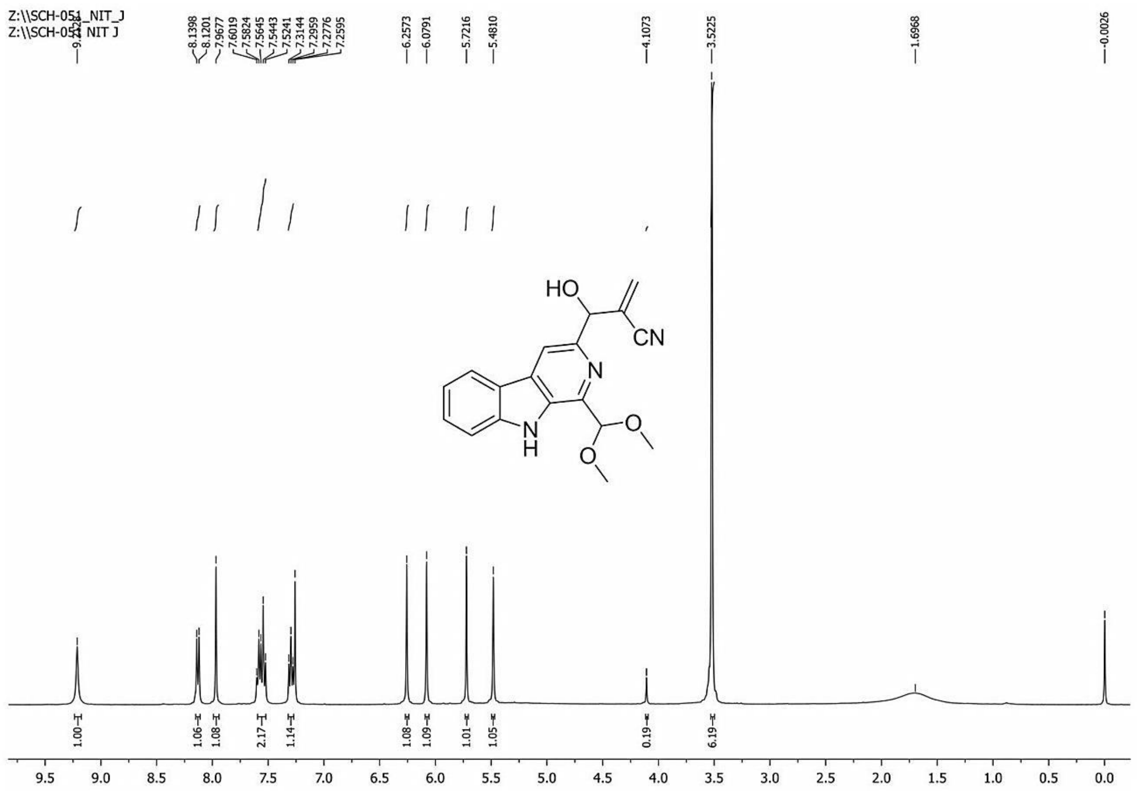

Fig. S3. ¹H NMR of 2-((1-(dimethoxymethyl)-9*H*-pyrido[3,4-*b*]indol-3-yl)(hydroxy)-methyl)acrylonitrile (**7aA**).

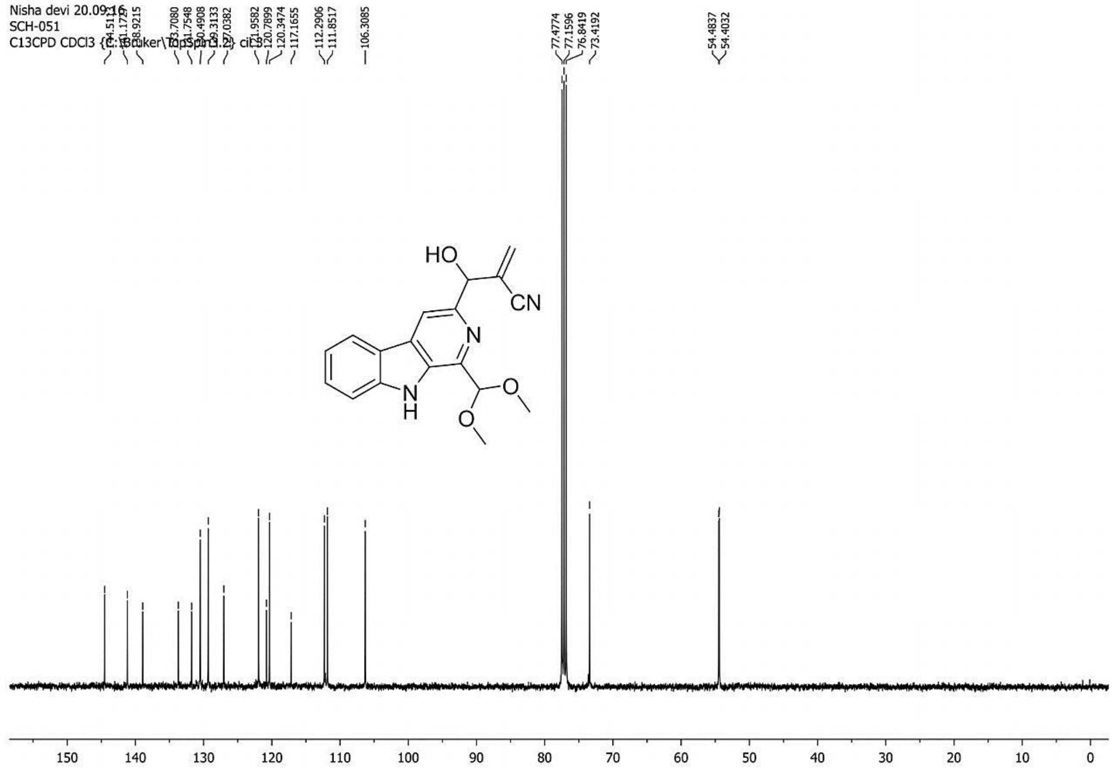

Fig. S4. ¹³C NMR of 2-((1-(dimethoxymethyl)-9*H*-pyrido[3,4-*b*]indol-3-yl)(hydroxy)-methyl)acrylonitrile (**7aA**).

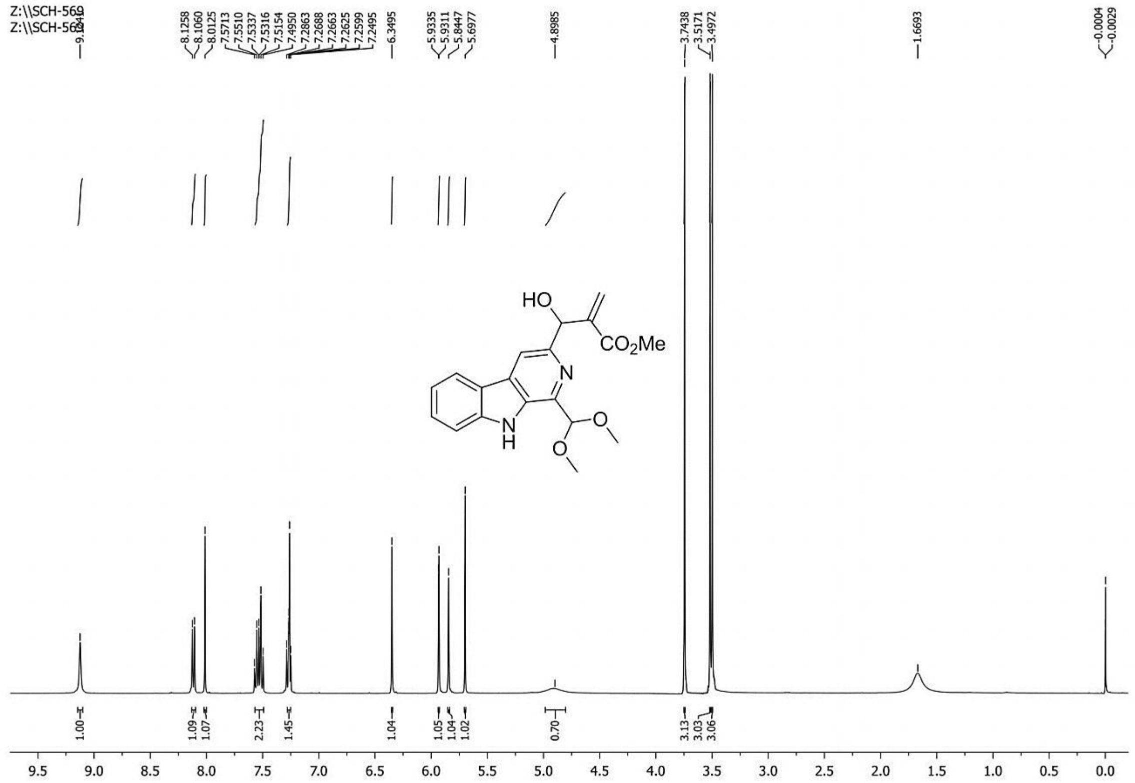

Fig. S5. ^{1}H NMR of methyl 2-((1-(dimethoxymethyl)-9*H*-pyrido[3,4-*b*]indol-3-yl)(hydroxy)methyl)acrylate (**7aB**).

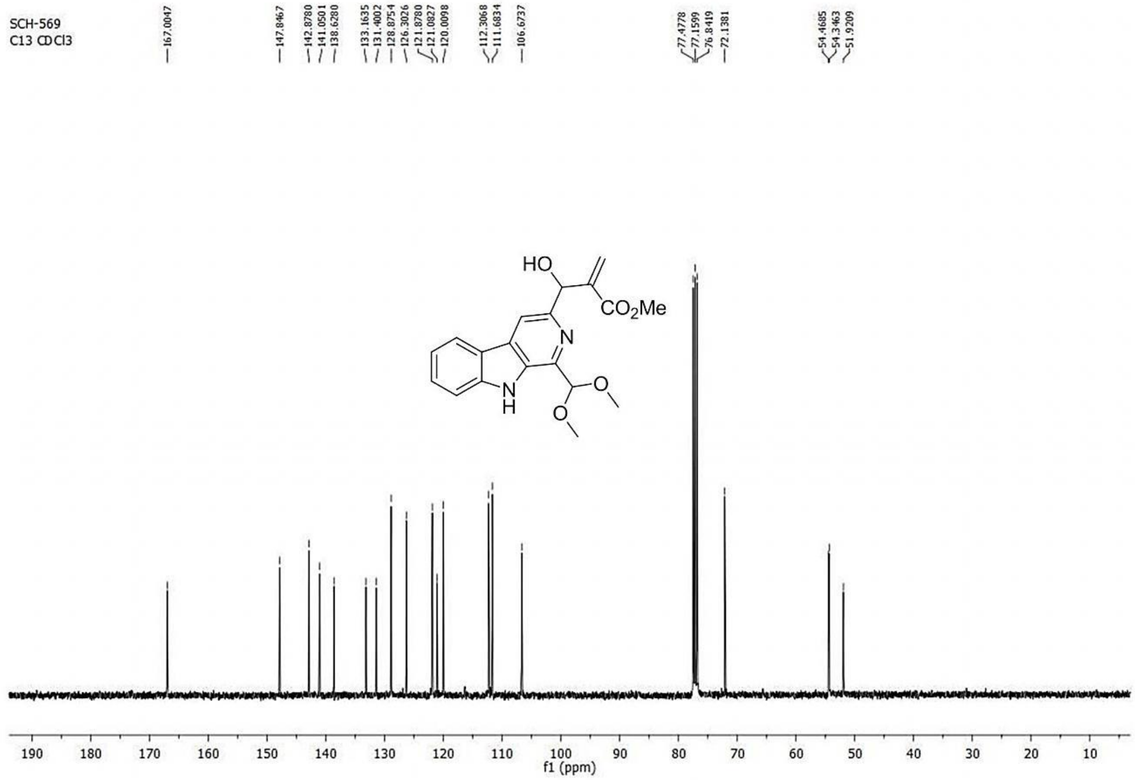

Fig. S6. ^{13}C NMR of methyl 2-((1-(dimethoxymethyl)-9*H*-pyrido[3,4-*b*]indol-3-yl)(hydroxy)methyl)acrylate (**7aB**).

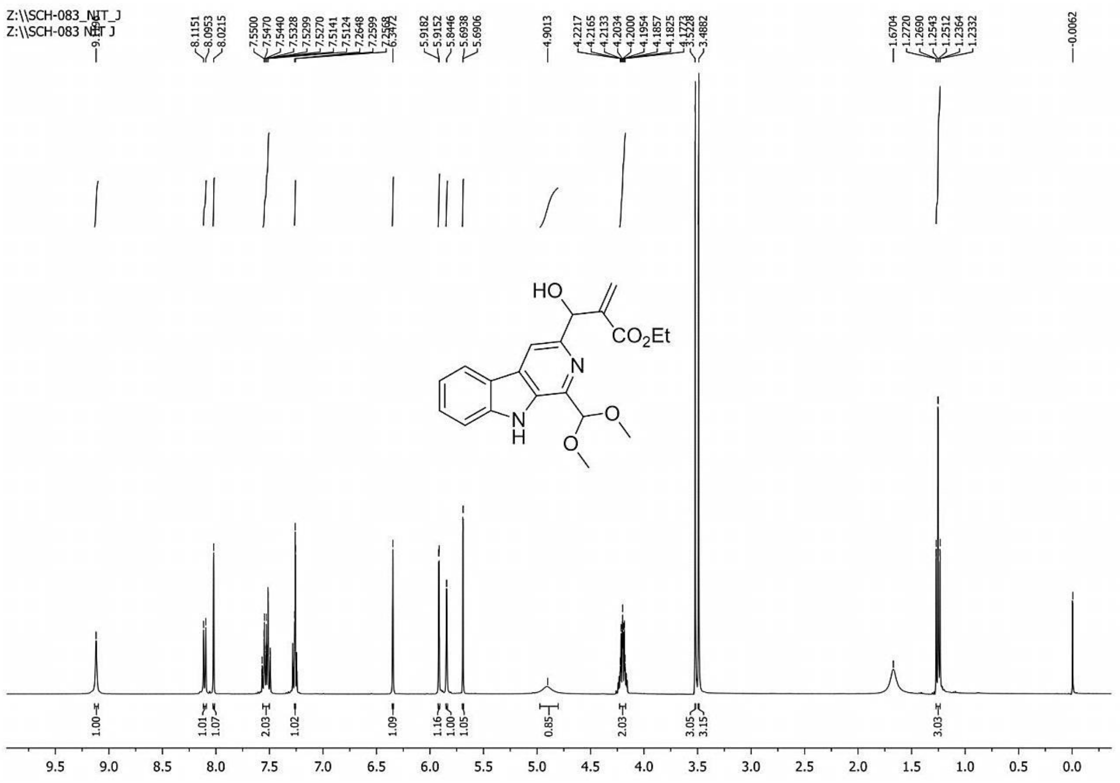

Fig. S7. ^1H NMR of ethyl 2-((1-(dimethoxymethyl)-9*H*-pyrido[3,4-*b*]indol-3-yl)(hydroxy)methyl)acrylate (**7aC**).

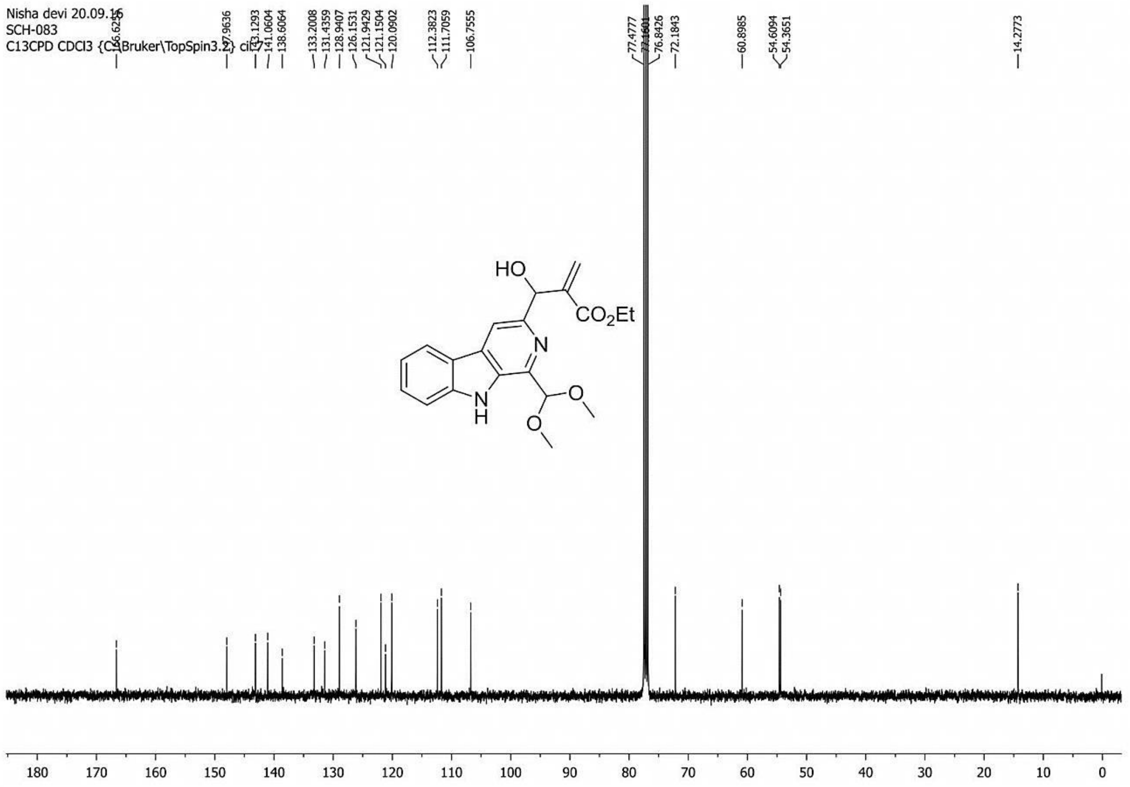

Fig. S8. ^{13}C-NMR of ethyl 2-((1-(dimethoxymethyl)-9*H*-pyrido[3,4-*b*]indol-3-yl)(hydroxy)methyl)acrylate (**7aC**).

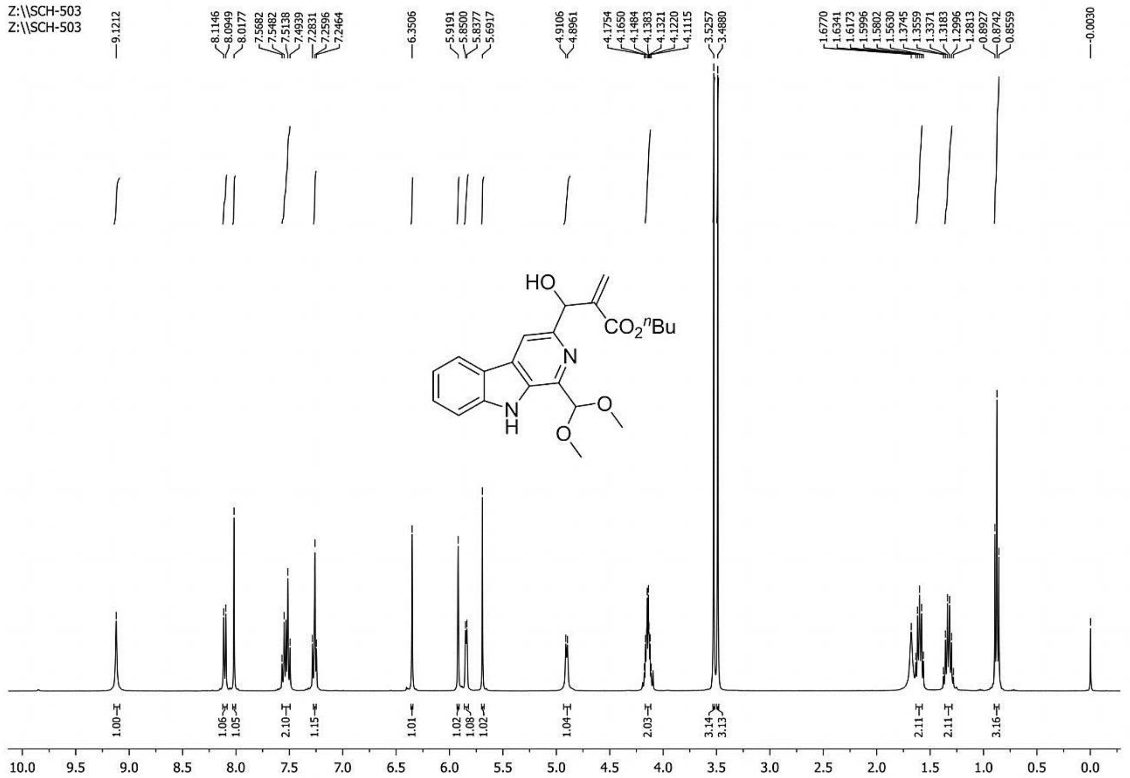

Fig. S9. ^{1}H NMR of butyl 2-((1-(dimethoxymethyl)-9*H*-pyrido[3,4-*b*]indol-3-yl)(hydroxy)methyl)acrylate (**7aD**).

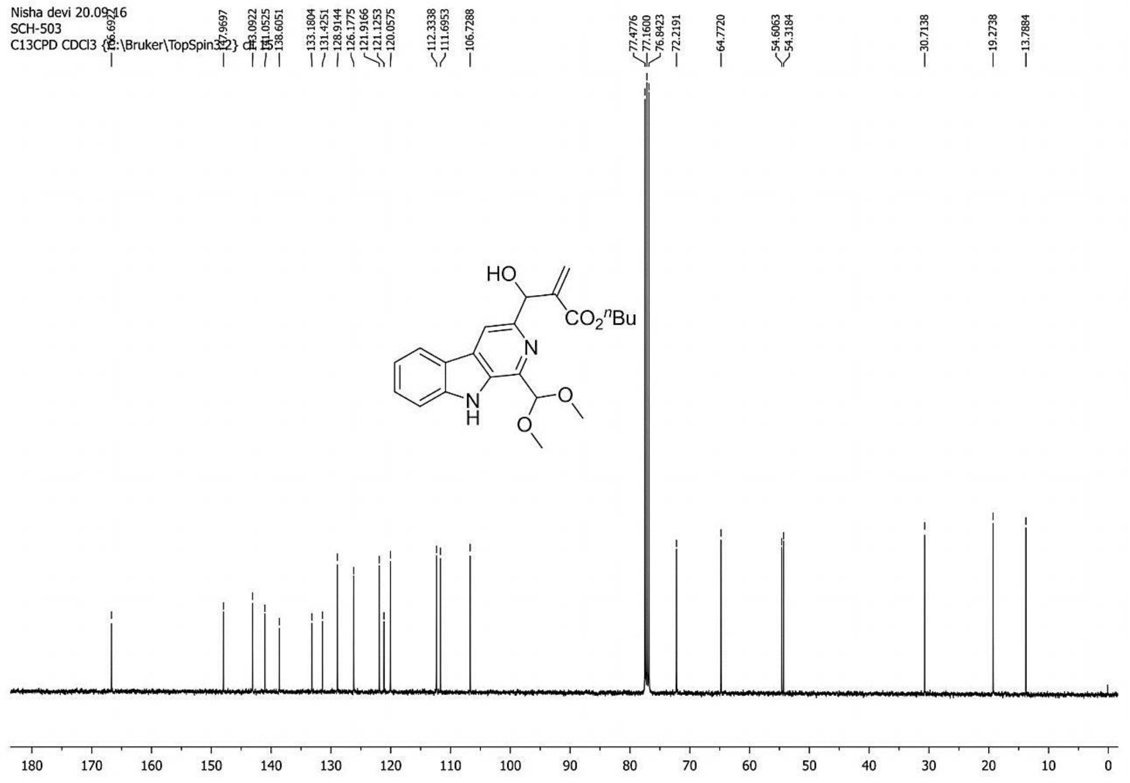

Fig. S10. ^{13}C NMR of butyl 2-((1-(dimethoxymethyl)-9*H*-pyrido[3,4-*b*]indol-3-yl)(hydroxy)methyl)acrylate (**7aD**).

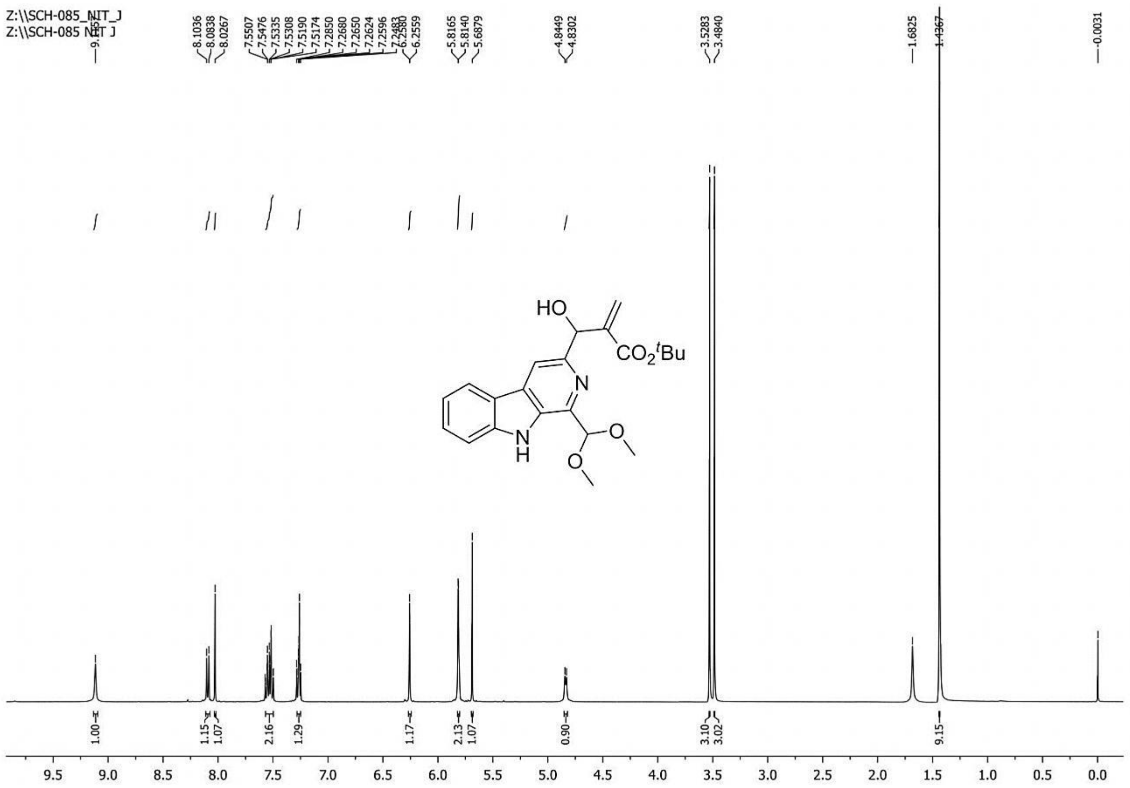

Fig. S11. ^{1}H NMR of *tert*-butyl 2-((1-(dimethoxymethyl)-9*H*-pyrido[3,4-*b*]indol-3-yl)(hydroxy)methyl)acrylate (**7aE**).

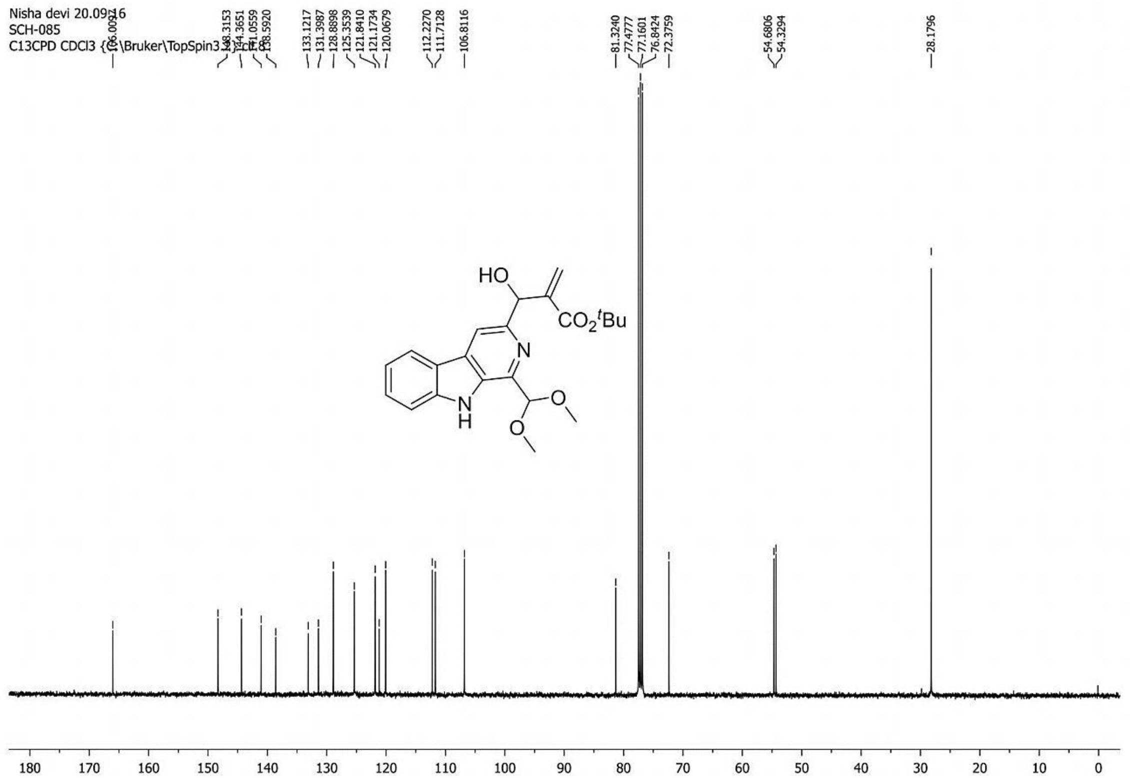

Fig. S12. ^{13}C NMR of *tert*-butyl 2-((1-(dimethoxymethyl)-9*H*-pyrido[3,4-*b*]indol-3-yl)(hydroxy)methyl)acrylate (**7aE**).

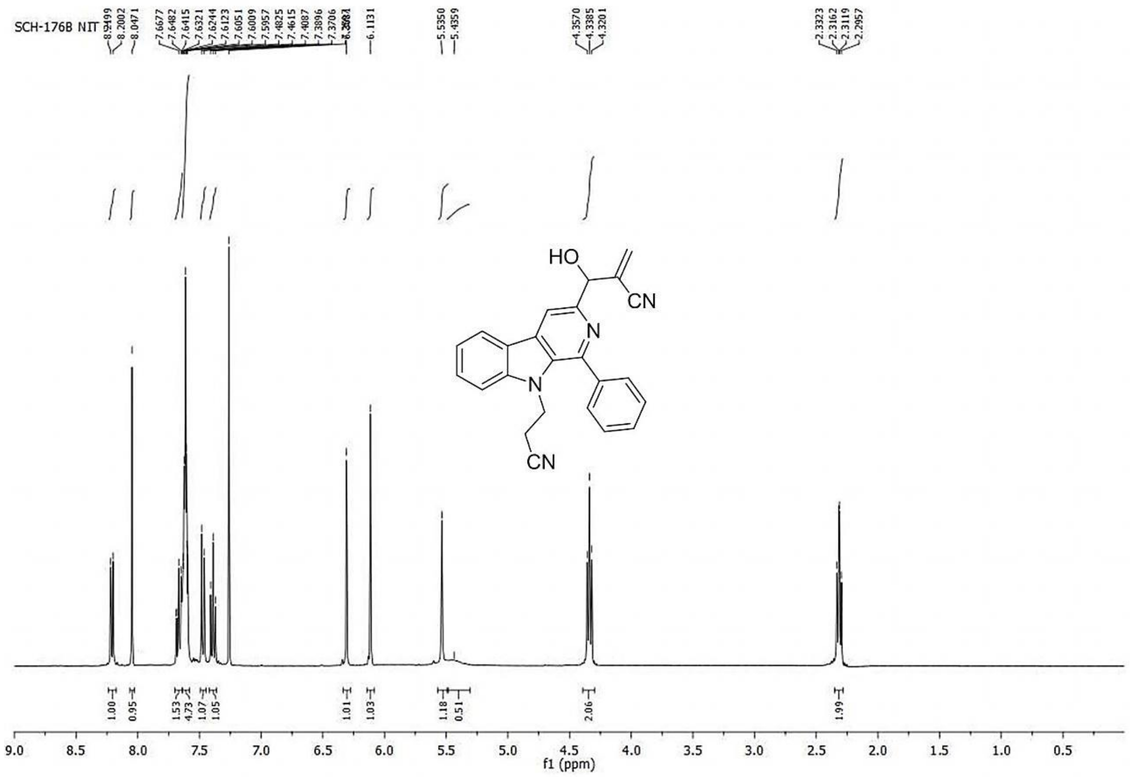

Fig. S13. ¹H NMR of 2-((9-(2-cyanoethyl)-1-phenyl-9*H*-pyrido[3,4-*b*]indol-3-yl)(hydroxy)methyl)acrylonitrile (**8bA**).

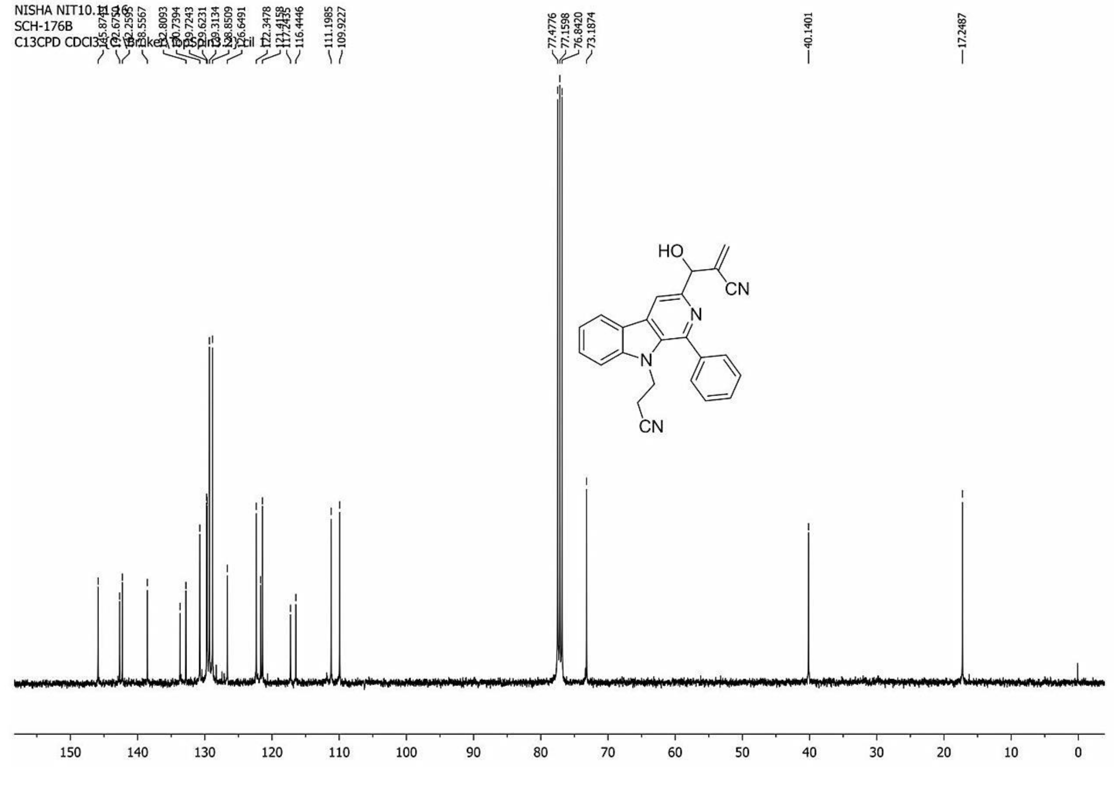

Fig. S14. ¹³C NMR of 2-((9-(2-cyanoethyl)-1-phenyl-9*H*-pyrido[3,4-*b*]indol-3-yl)(hydroxy)methyl)acrylonitrile (**8bA**).

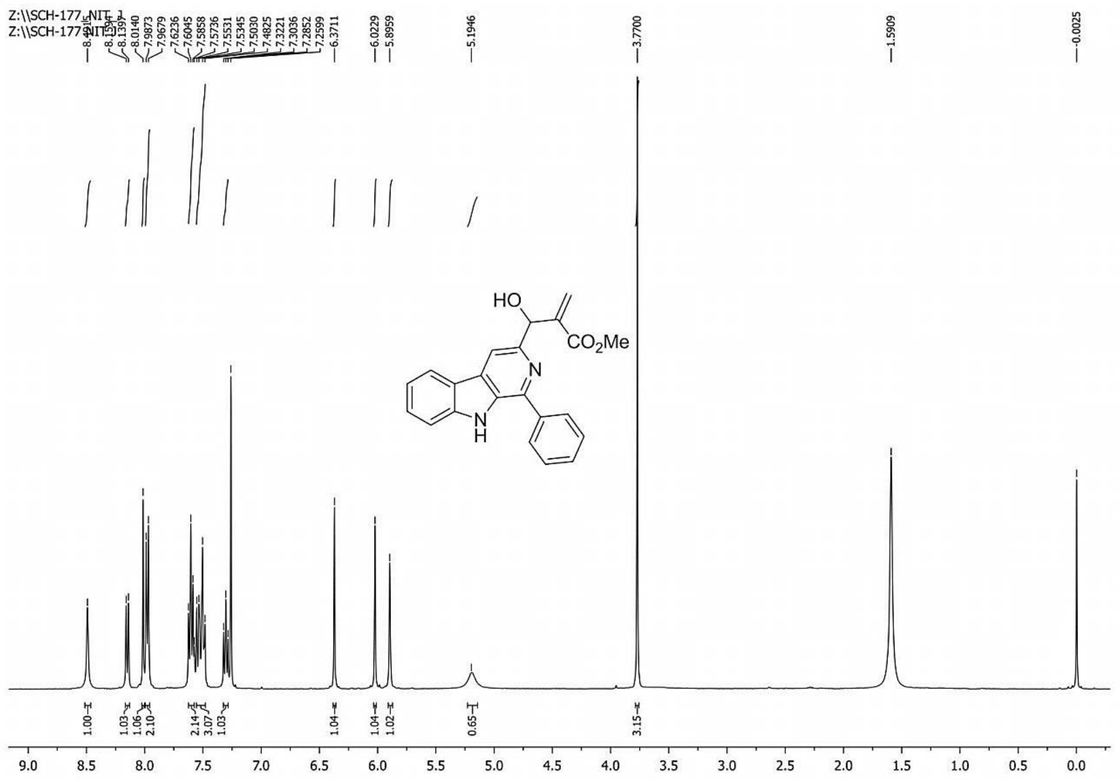

Fig. S15. ^{1}H NMR of methyl 2-(hydroxy(1-phenyl-9*H*-pyrido[3,4-*b*]indol-3-yl)methyl)acrylate (**7bB**).

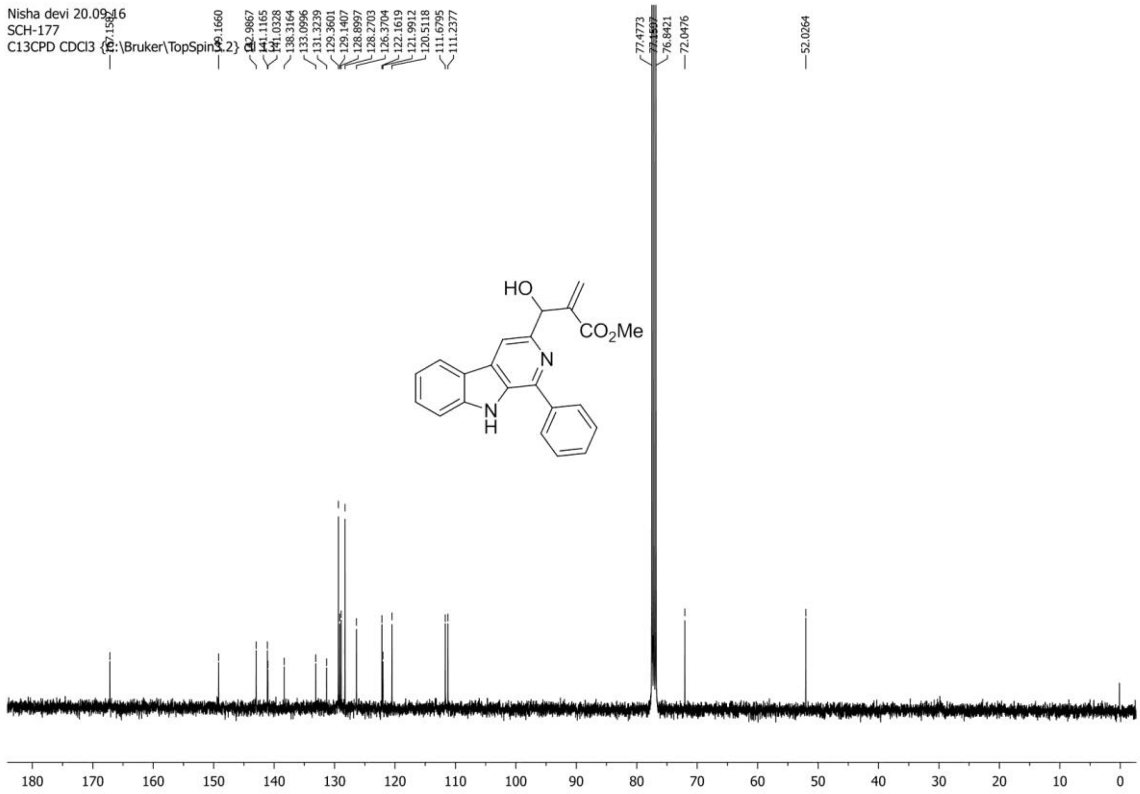

Fig. S16. ^{13}C NMR of methyl 2-(hydroxy(1-phenyl-9*H*-pyrido[3,4-*b*]indol-3-yl)methyl)acrylate (**7bB**).

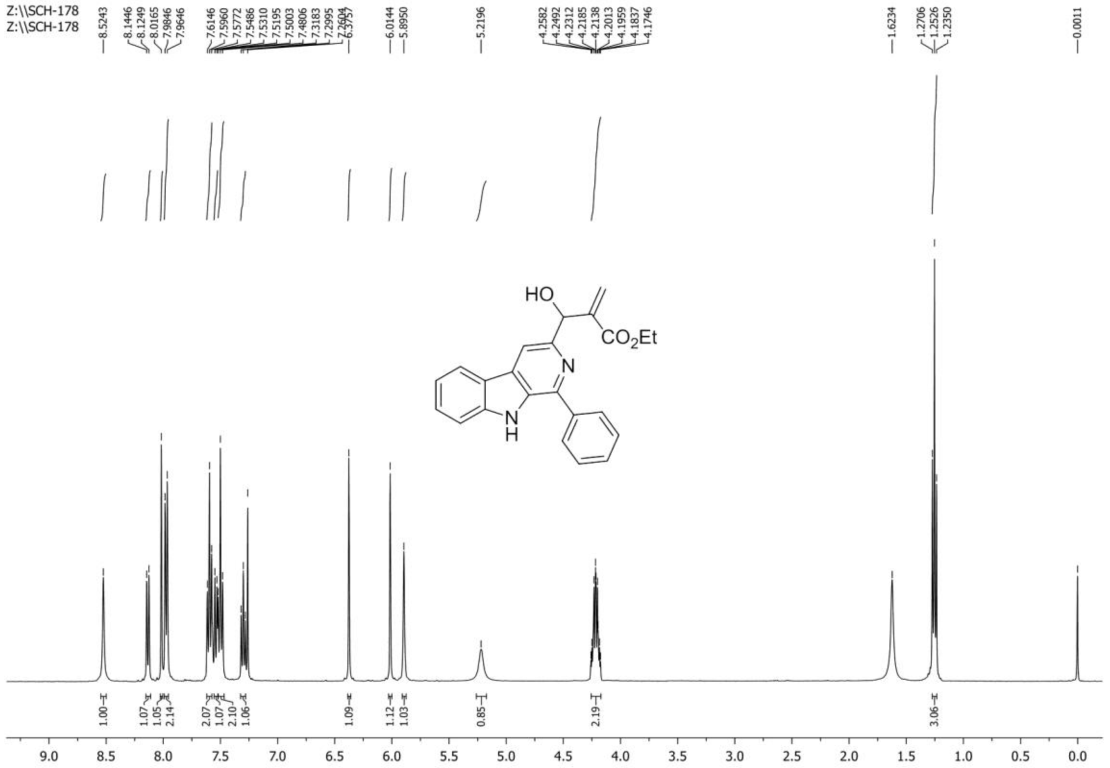

Fig. S17. ^{1}H NMR of ethyl 2-(hydroxy(1-phenyl-9*H*-pyrido[3,4-*b*]indol-3-yl)methyl)acrylate (**7bC**).

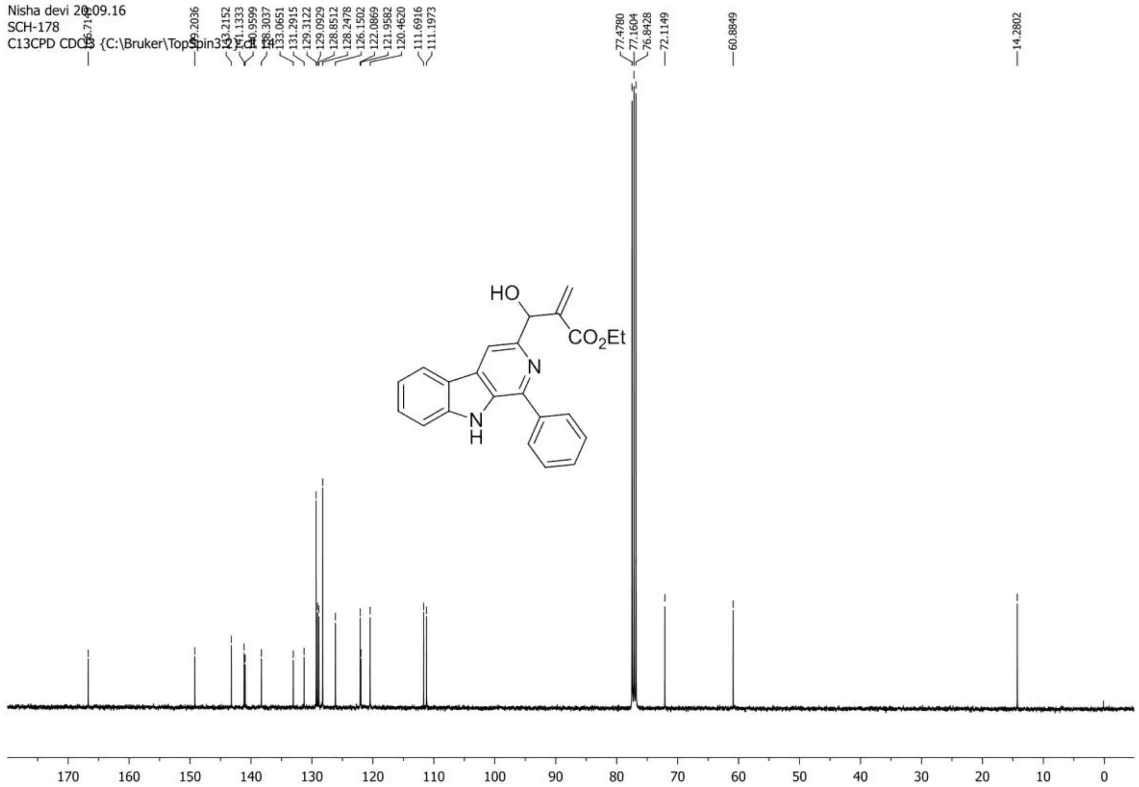

Fig. S18. ^{13}C NMR of ethyl 2-(hydroxy(1-phenyl-9*H*-pyrido[3,4-*b*]indol-3-yl)methyl)acrylate (**7bC**).

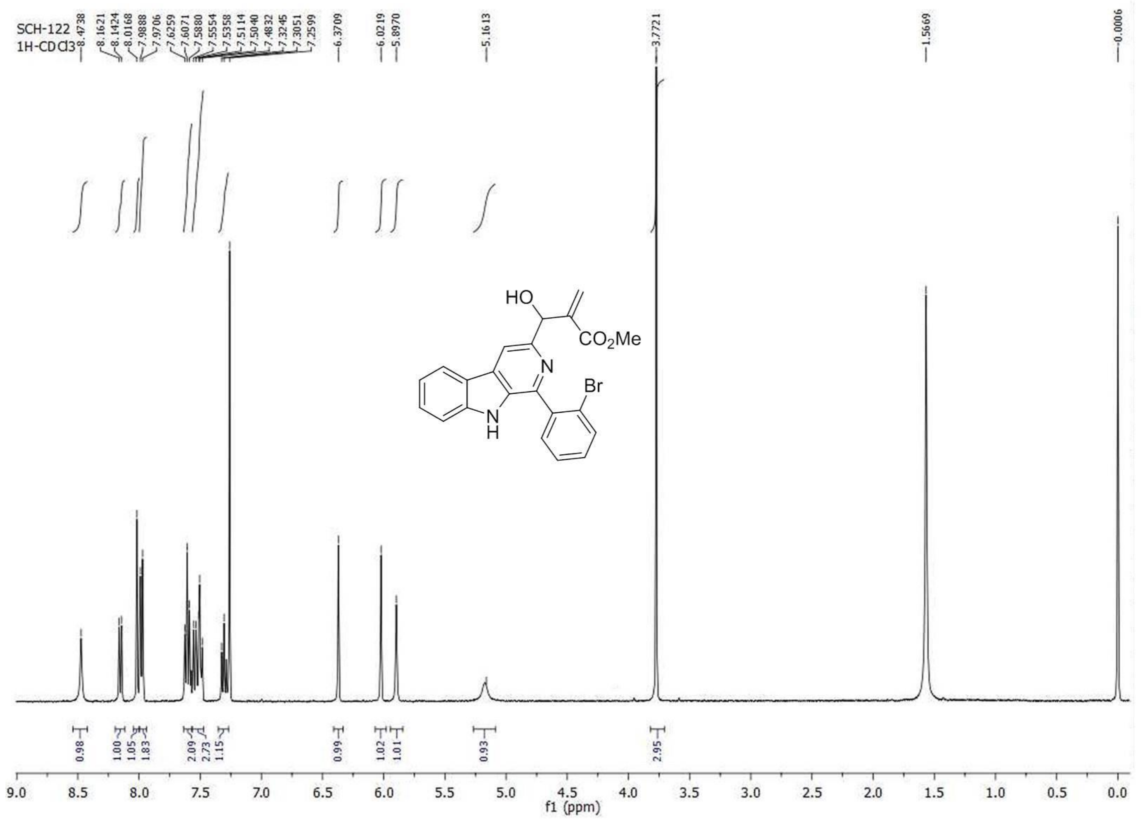

Fig. S19. ^{1}H NMR of methyl 2-((1-(2-bromophenyl)-9*H*-pyrido[3,4-*b*]indol-3-yl)(hydroxy)methyl)acrylate (**7cB**).

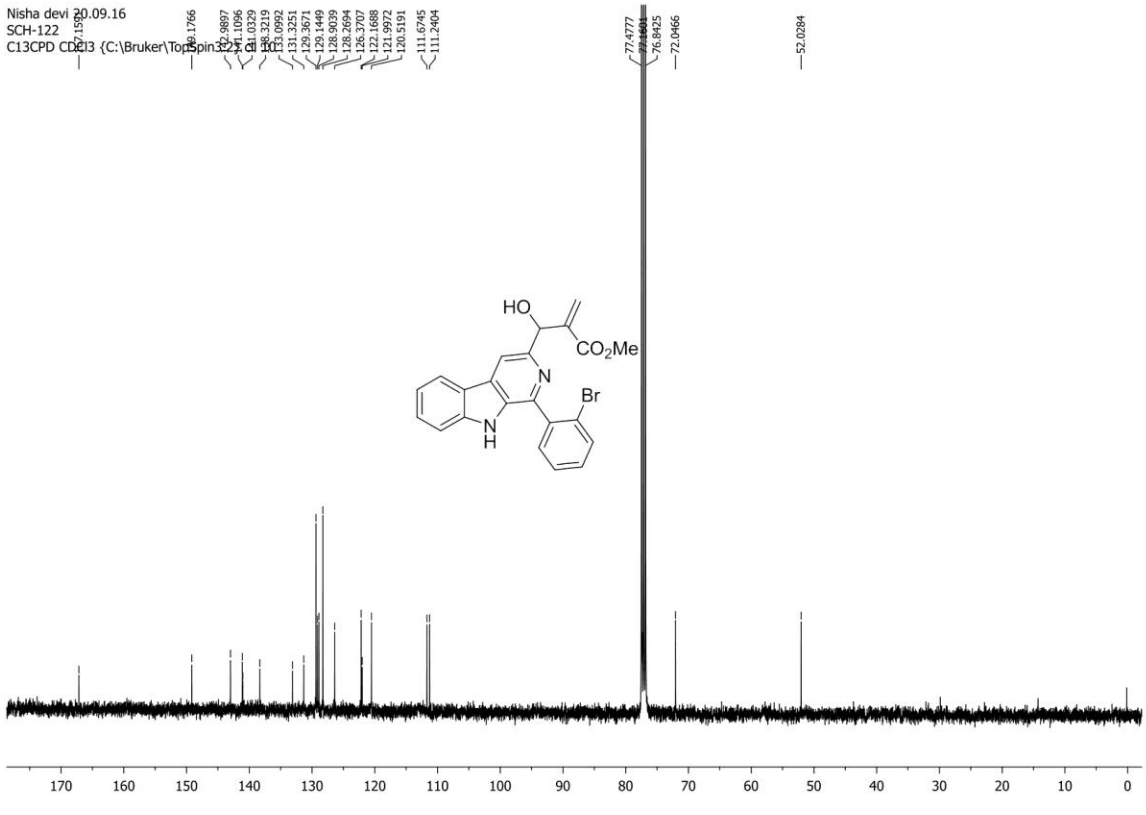

Fig. S20. ^{13}C NMR of methyl 2-((1-(2-bromophenyl)-9*H*-pyrido[3,4-*b*]indol-3-yl)(hydroxy)methyl)acrylate (**7cB**).

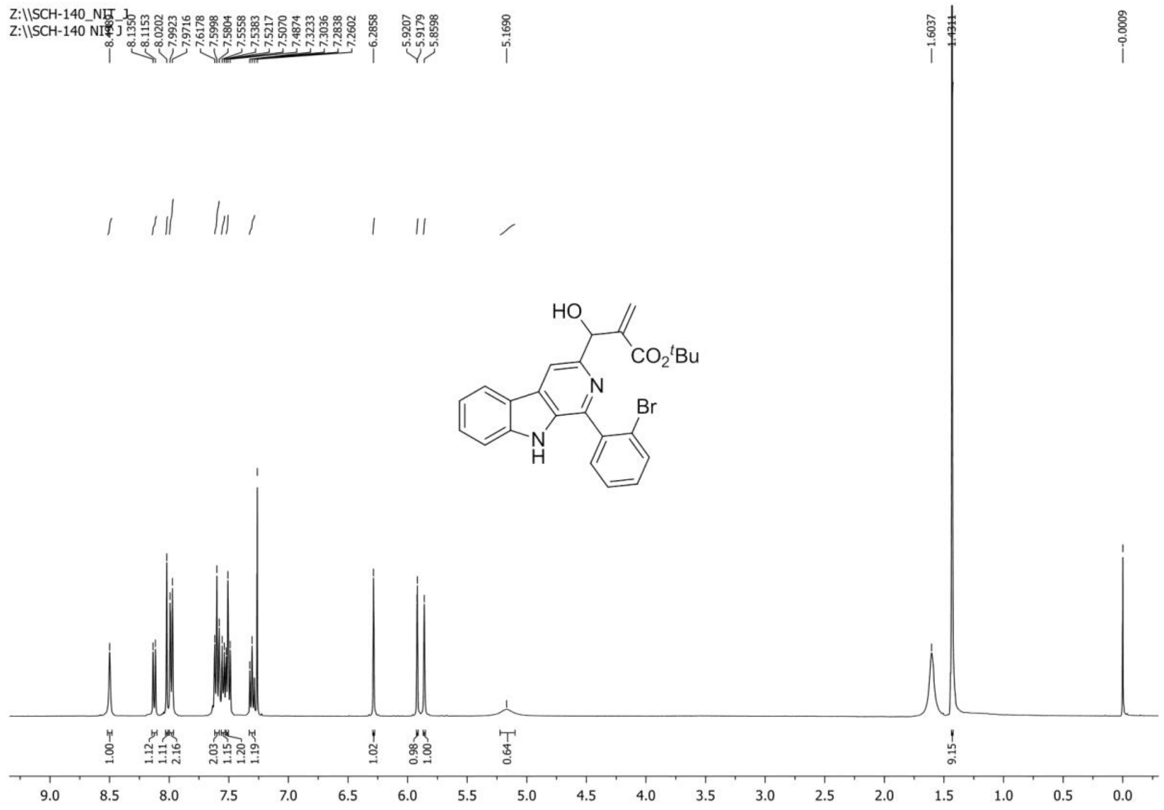

Fig. S21. ^{1}H NMR of *tert*-butyl 2-((1-(2-bromophenyl)-9*H*-pyrido[3,4-*b*]indol-3-yl)(hydroxy)methyl)acrylate (**7cE**).

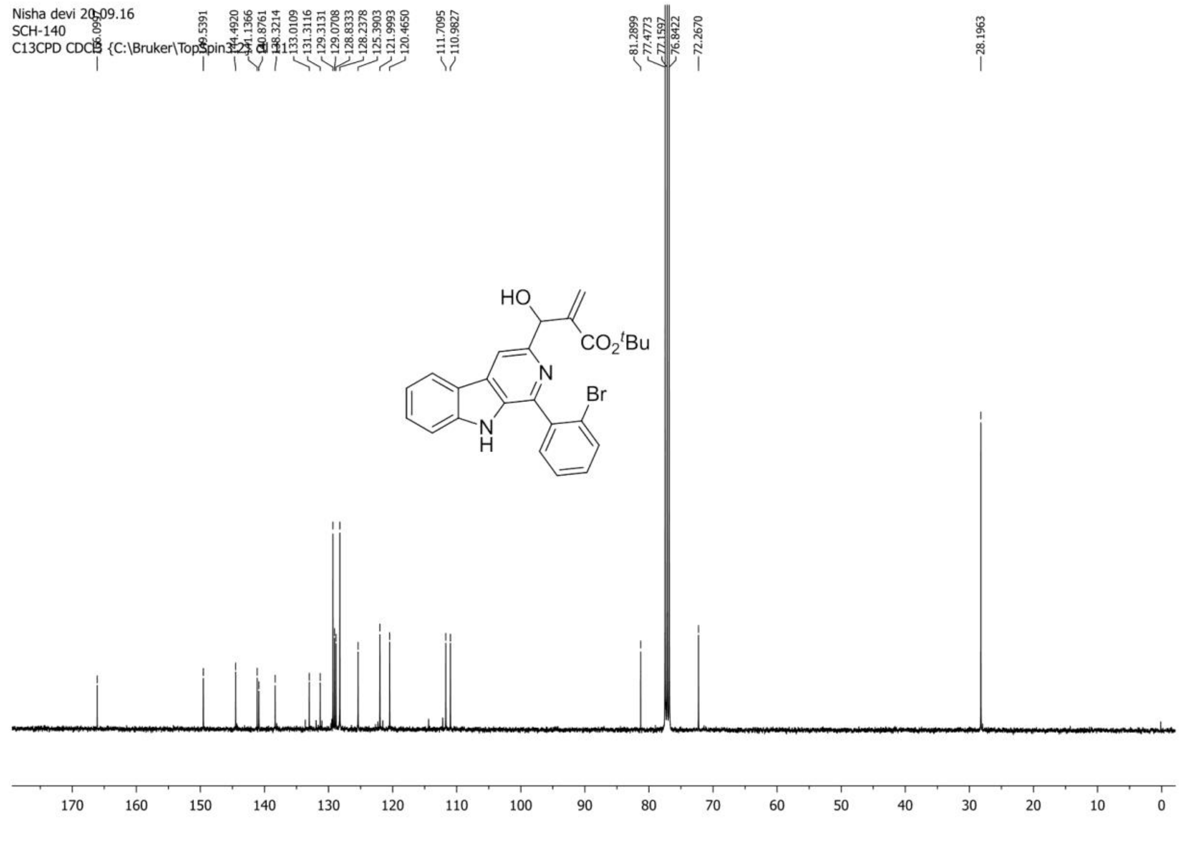

Fig. S22. ^{13}C NMR of *tert*-butyl 2-((1-(2-bromophenyl)-9*H*-pyrido[3,4-*b*]indol-3-yl)(hydroxy)methyl)acrylate (**7cE**).

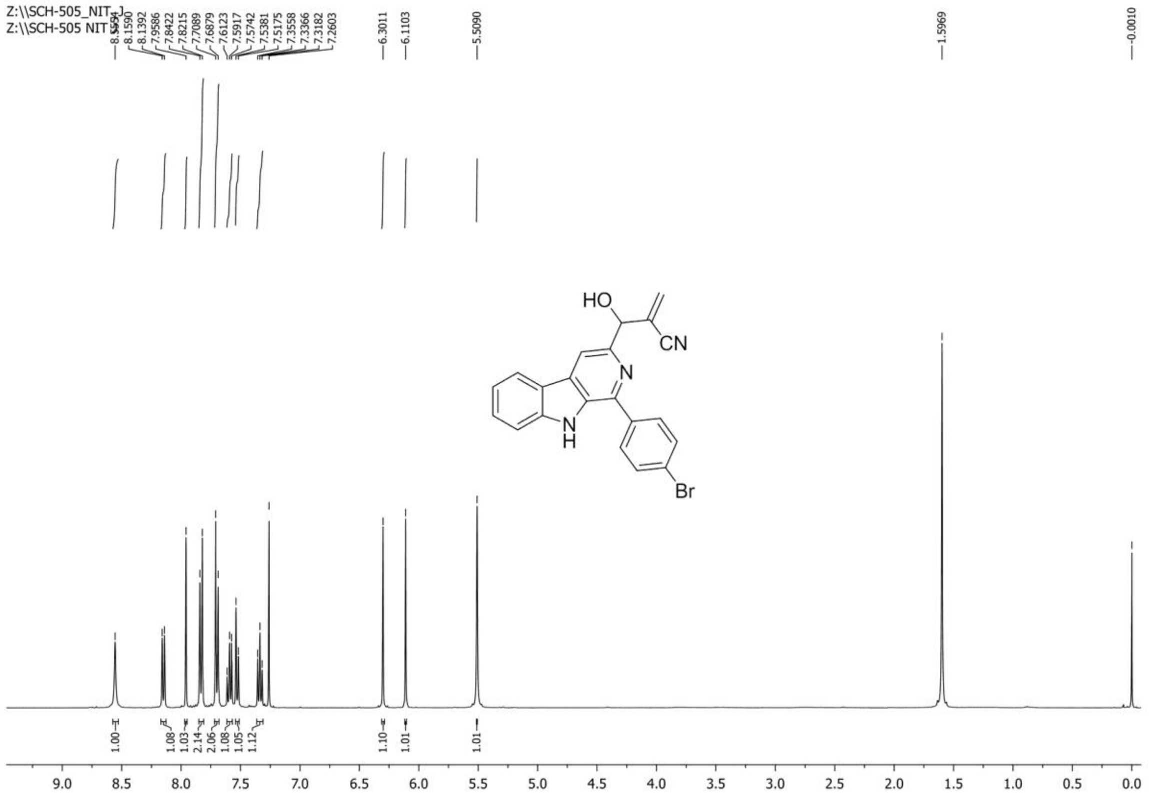

Fig. S23. ^{1}H NMR of 2-((1-(4-bromophenyl)-9*H*-pyrido[3,4-*b*]indol-3-yl)(hydroxy)methyl)acrylonitrile (**7dA**).

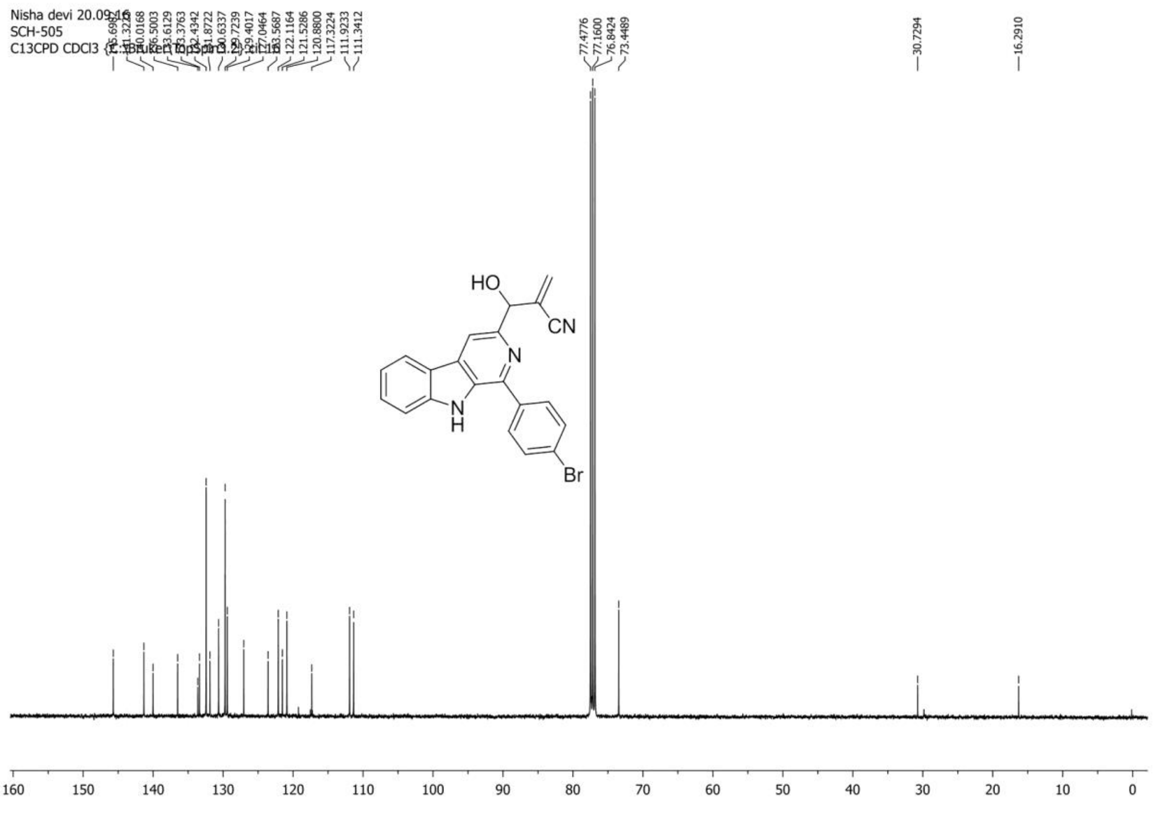

Fig. S24. ^{13}C NMR of 2-((1-(4-bromophenyl)-9*H*-pyrido[3,4-*b*]indol-3-yl)(hydroxy)methyl)acrylonitrile (**7dA**).

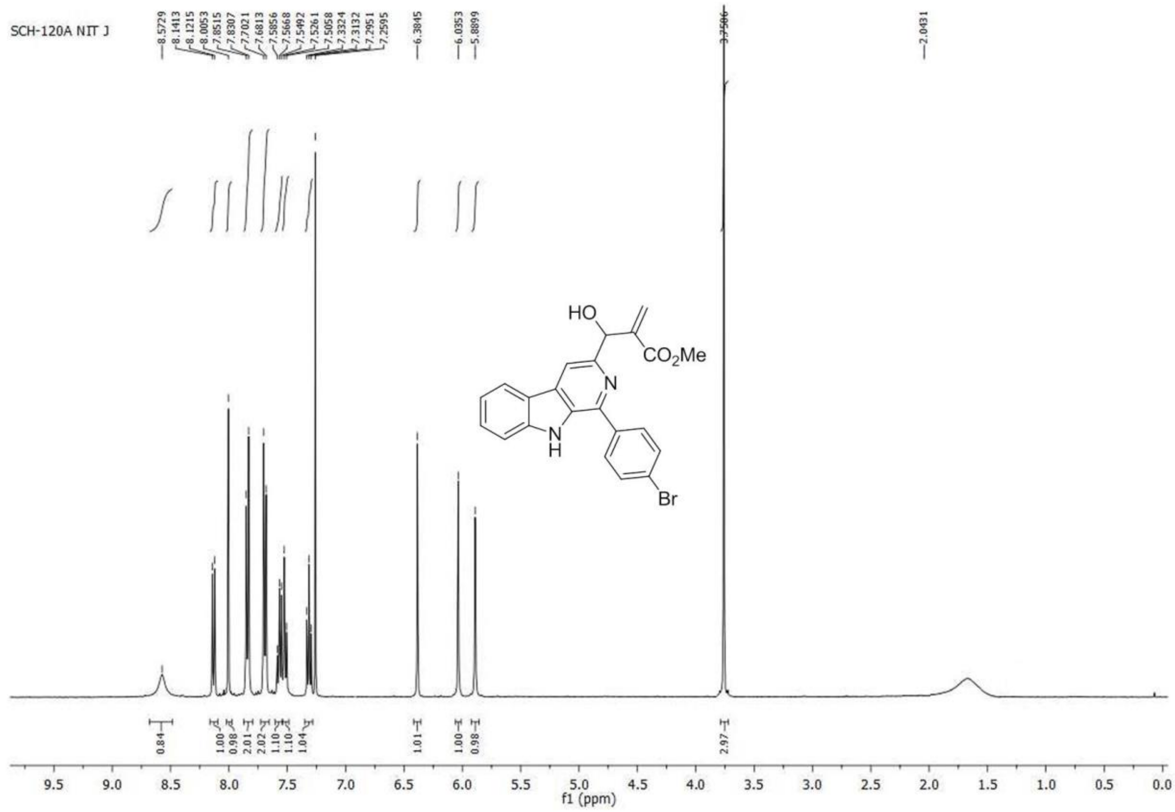

Fig. S25. ^{1}H NMR of methyl 2-((1-(4-bromophenyl)-9*H*-pyrido[3,4-*b*]indol-3-yl)(hydroxy)methyl)acrylate (**7dB**).

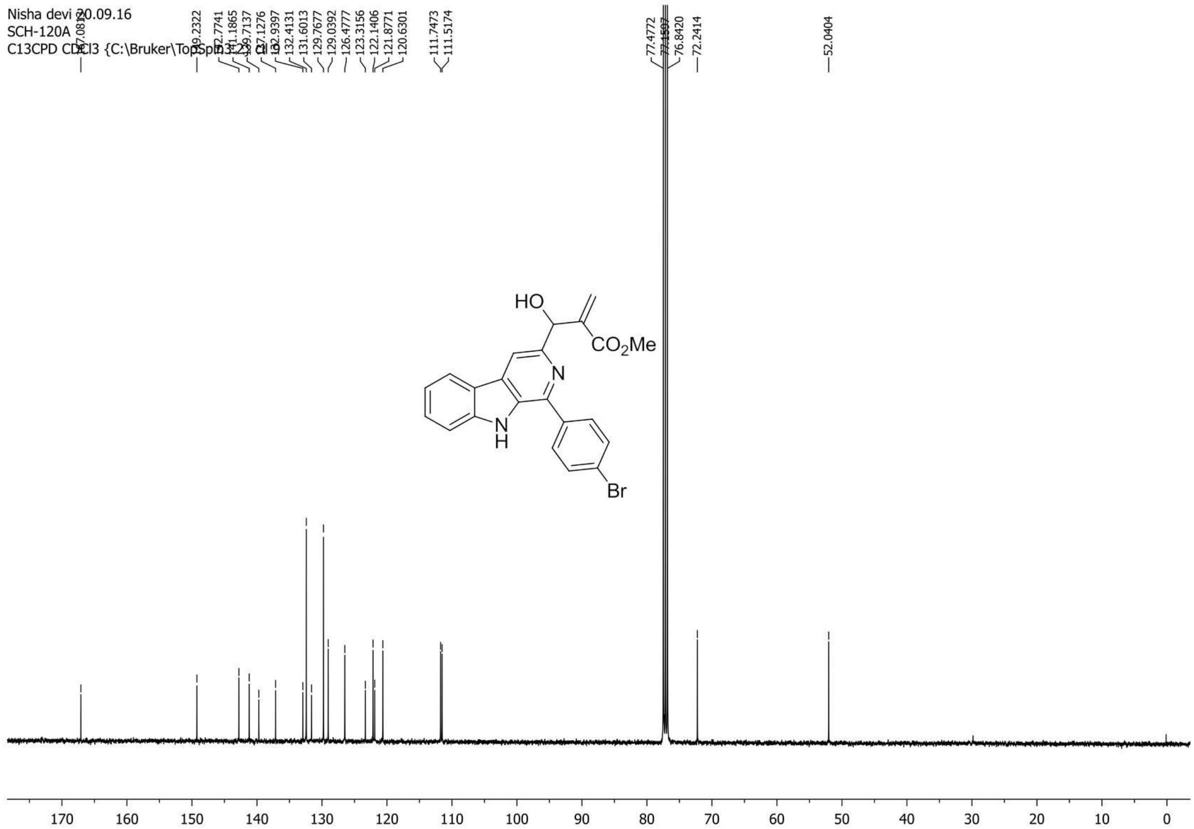

Fig. S26. ^{13}C NMR of methyl 2-((1-(4-bromophenyl)-9*H*-pyrido[3,4-*b*]indol-3-yl)(hydroxy)methyl)acrylate (**7dB**).

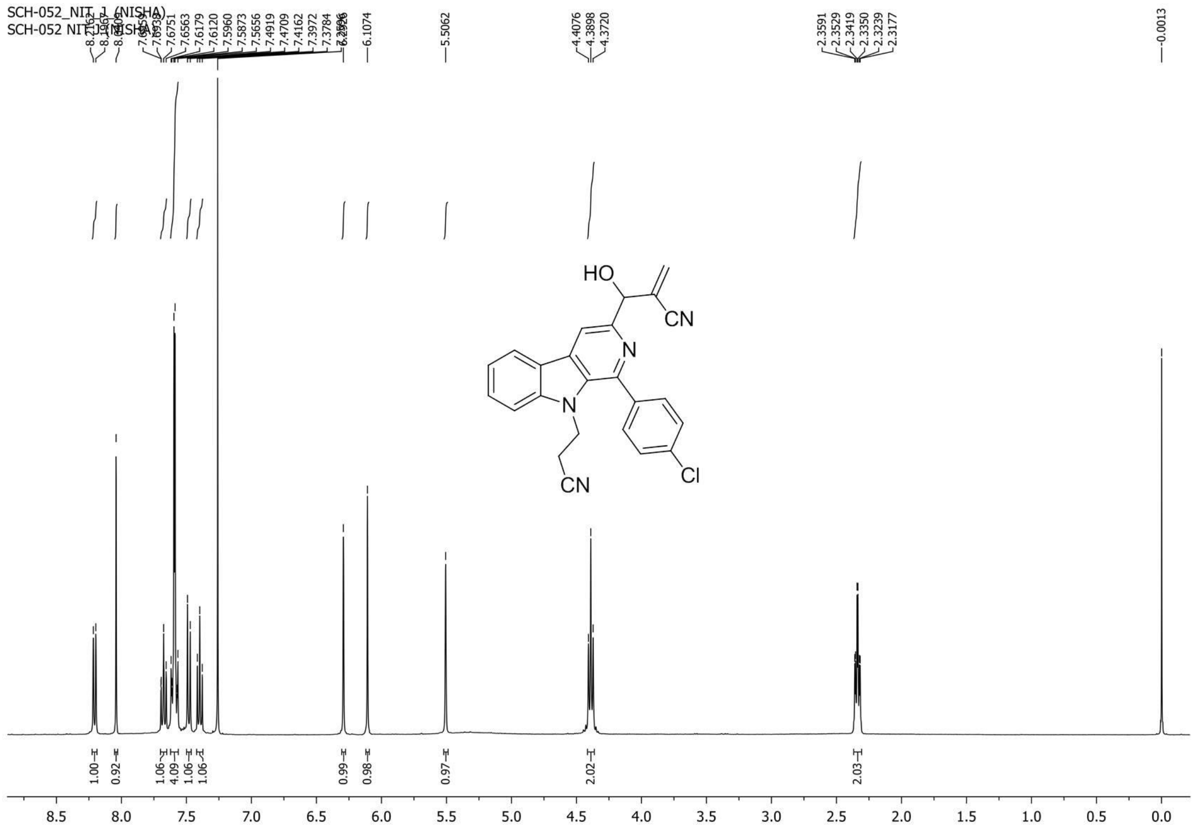

Fig. S27. ^{1}H NMR of 2-((1-(4-chlorophenyl)-9-(2-cyanoethyl)-9*H*-pyrido[3,4-*b*]indol-3-yl)(hydroxy)methyl) acrylonitrile (**8eA**).

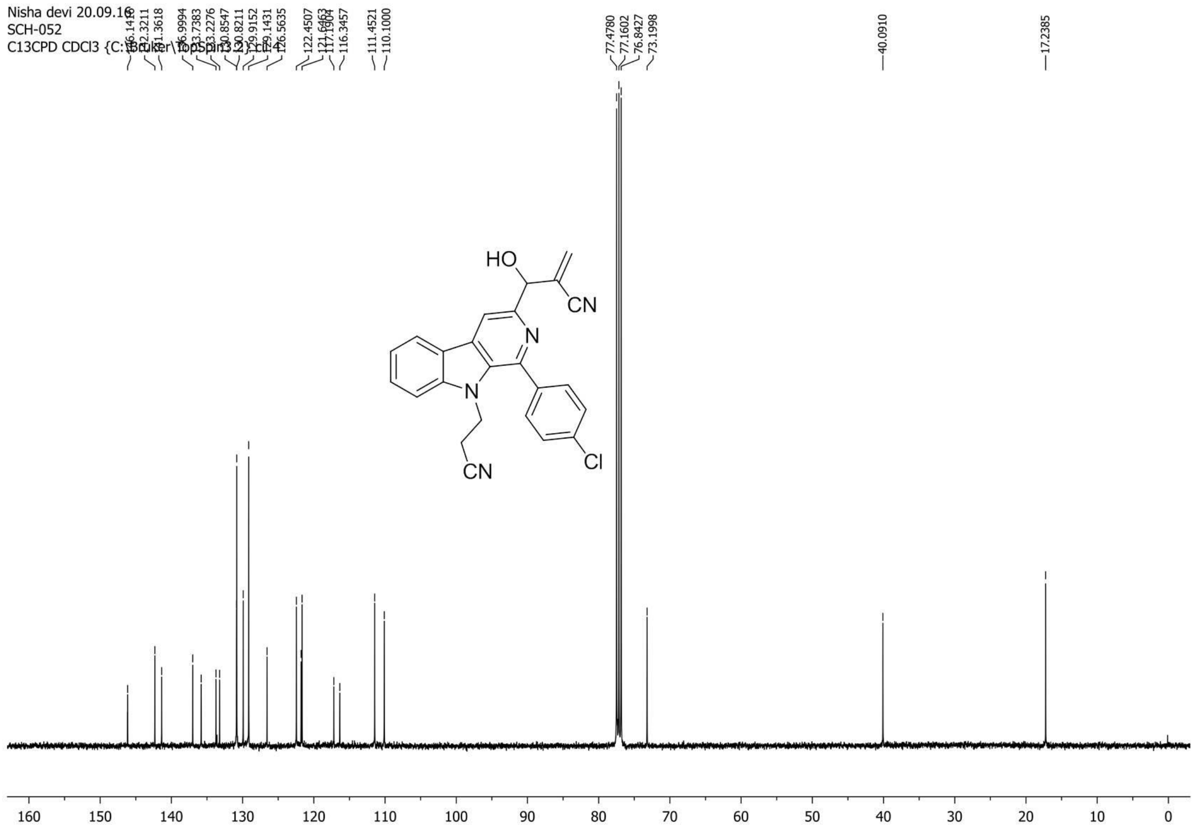

Fig. S28. ^{13}C NMR of 2-((1-(4-chlorophenyl)-9-(2-cyanoethyl)-9*H*-pyrido[3,4-*b*]indol-3-yl)(hydroxy)methyl) acrylonitrile (**8eA**).

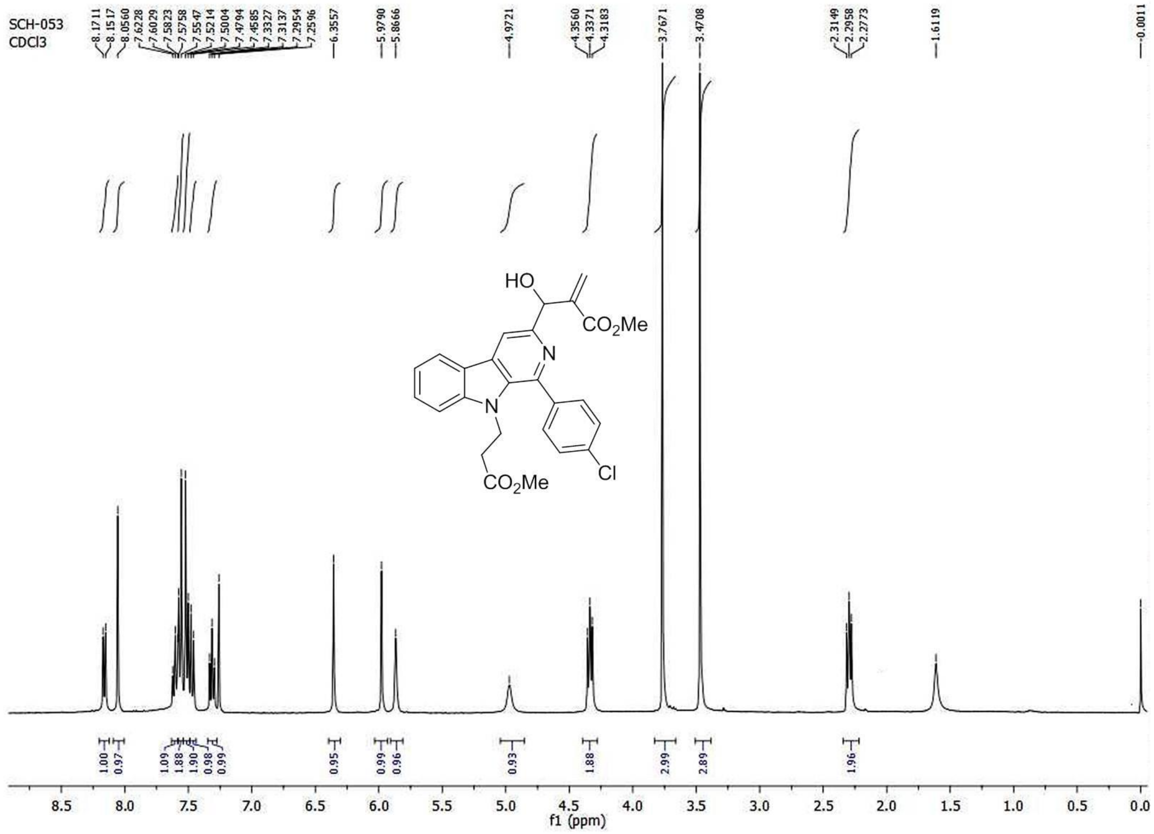

Fig. S29. ^{1}H NMR of methyl 2-((1-(4-chlorophenyl)-9-(3-methoxy-3-oxopropyl)-9*H*-pyrido[3,4-*b*]indol-3-yl)(hydroxy)methyl)acrylate (**8eB**).

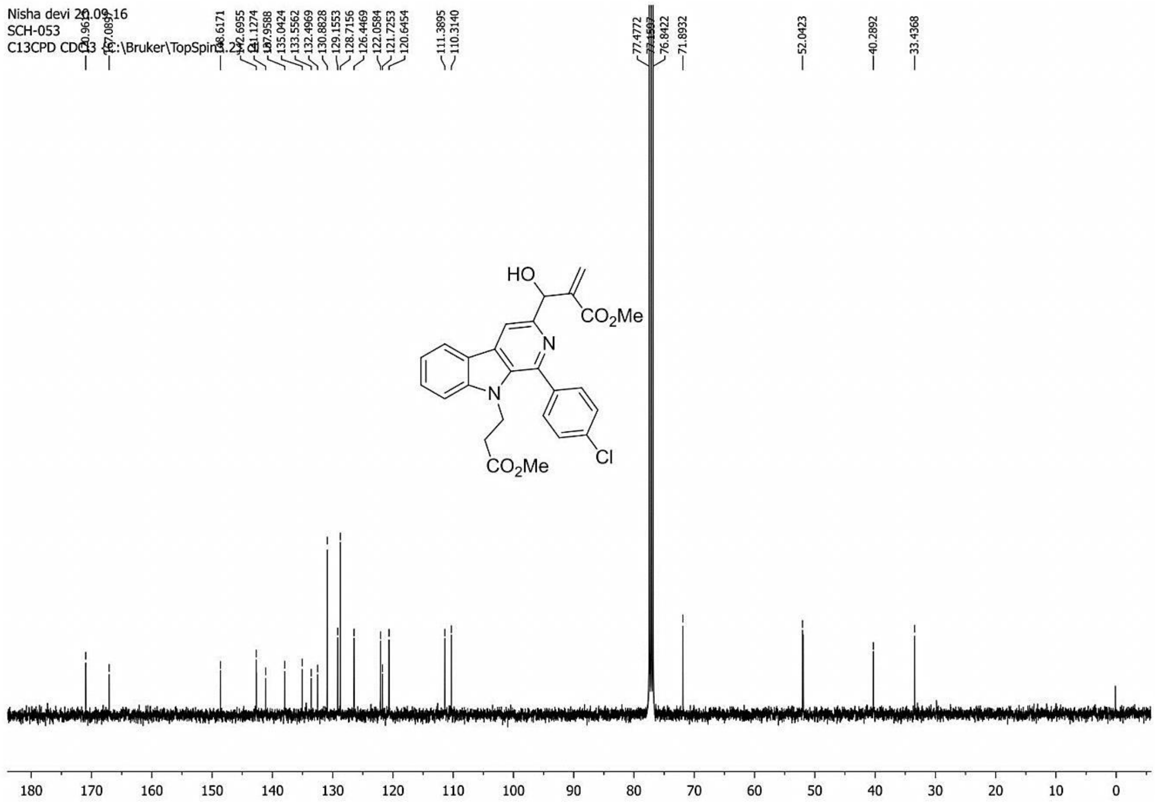

Fig. S30. ^{13}C NMR of methyl 2-((1-(4-chlorophenyl)-9-(3-methoxy-3-oxopropyl)-9*H*-pyrido[3,4-*b*]indol-3-yl)(hydroxy)methyl)acrylate (**8eB**).

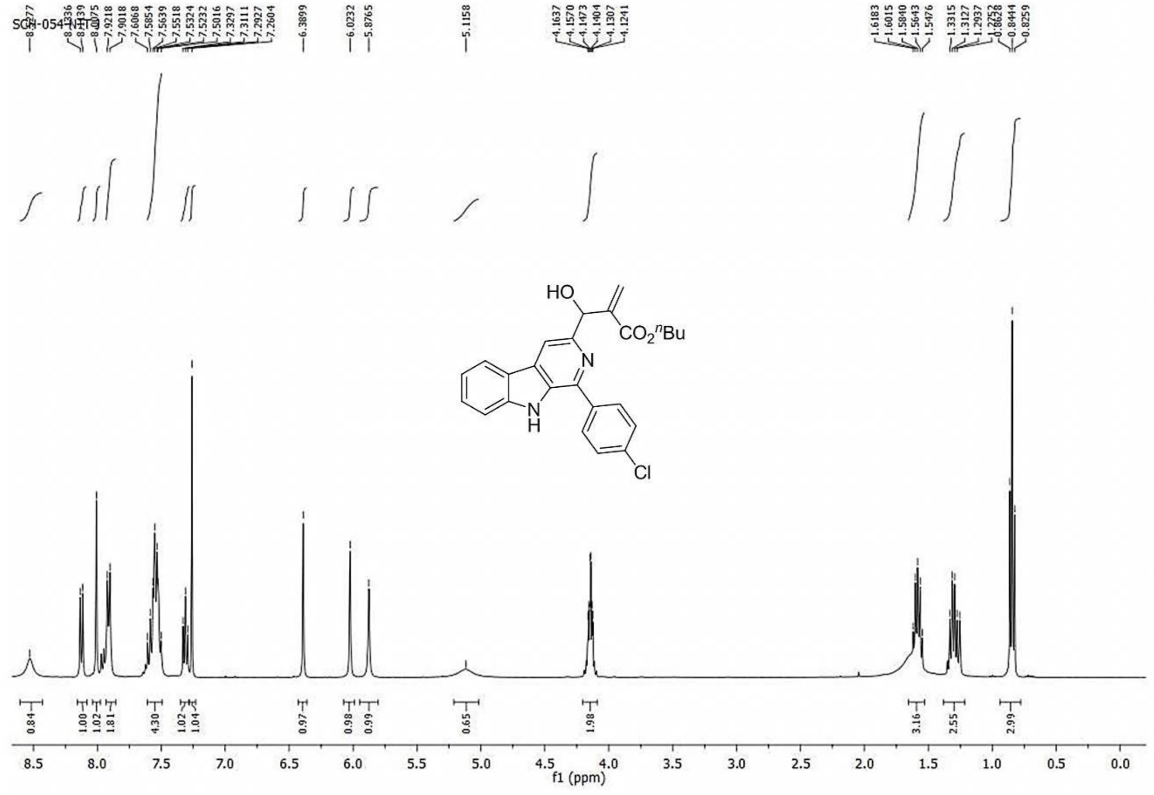

Fig. S31. ^{1}H NMR of butyl 2-((1-(4-chlorophenyl)-9*H*-pyrido[3,4-*b*]indol-3-yl)(hydroxy)methyl)acrylate (**7eD**).

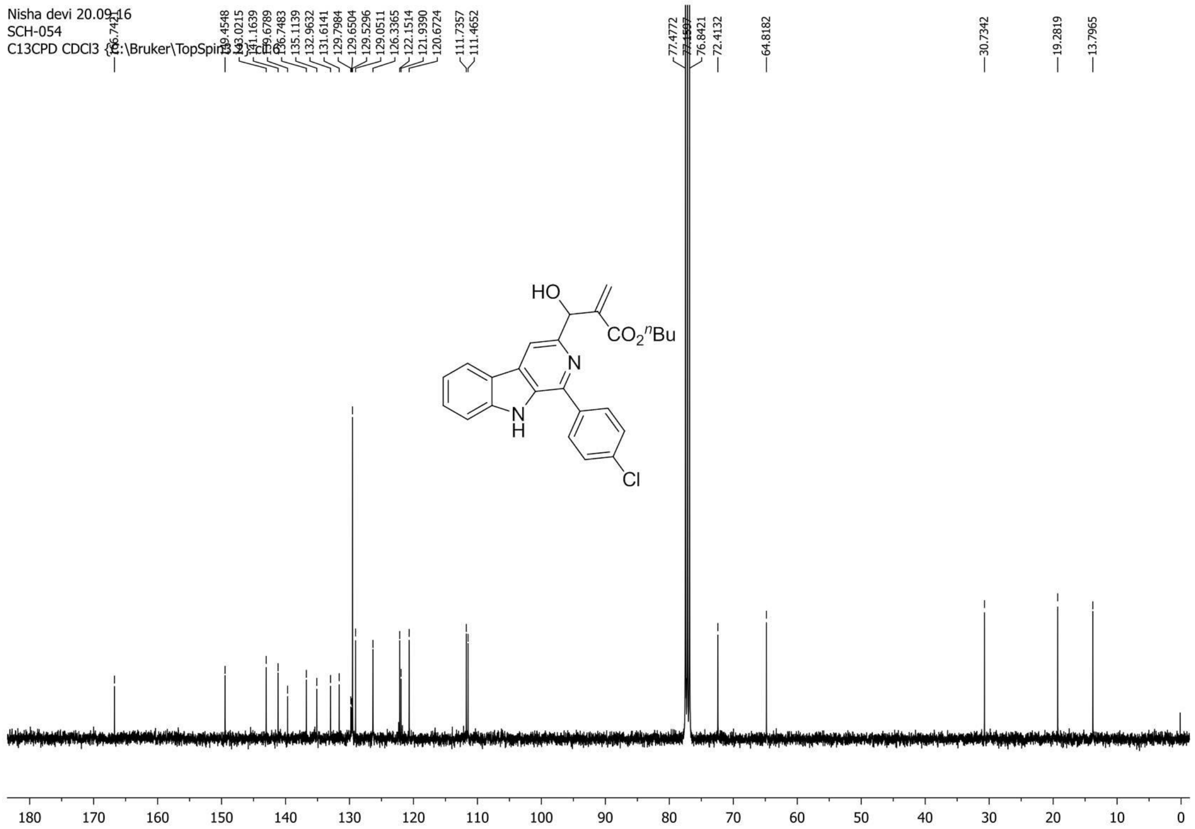

Fig. S32. ^{13}C NMR of butyl 2-((1-(4-chlorophenyl)-9*H*-pyrido[3,4-*b*]indol-3-yl)(hydroxy)methyl)acrylate (**7eD**).

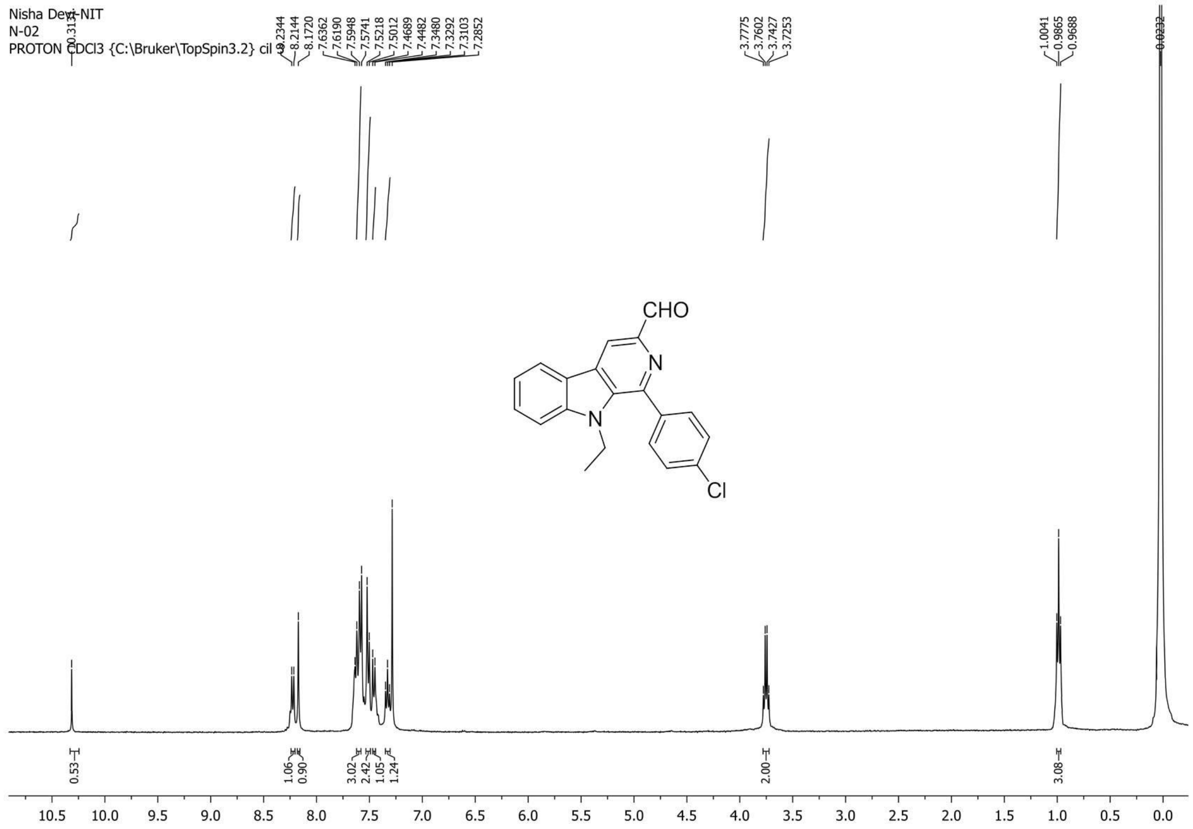

Fig. S33. ^{1}H NMR of 1-(4-chlorophenyl)-9-ethyl-9*H*-pyrido[3,4-*b*]indole-3-carbaldehyde (**9e**).

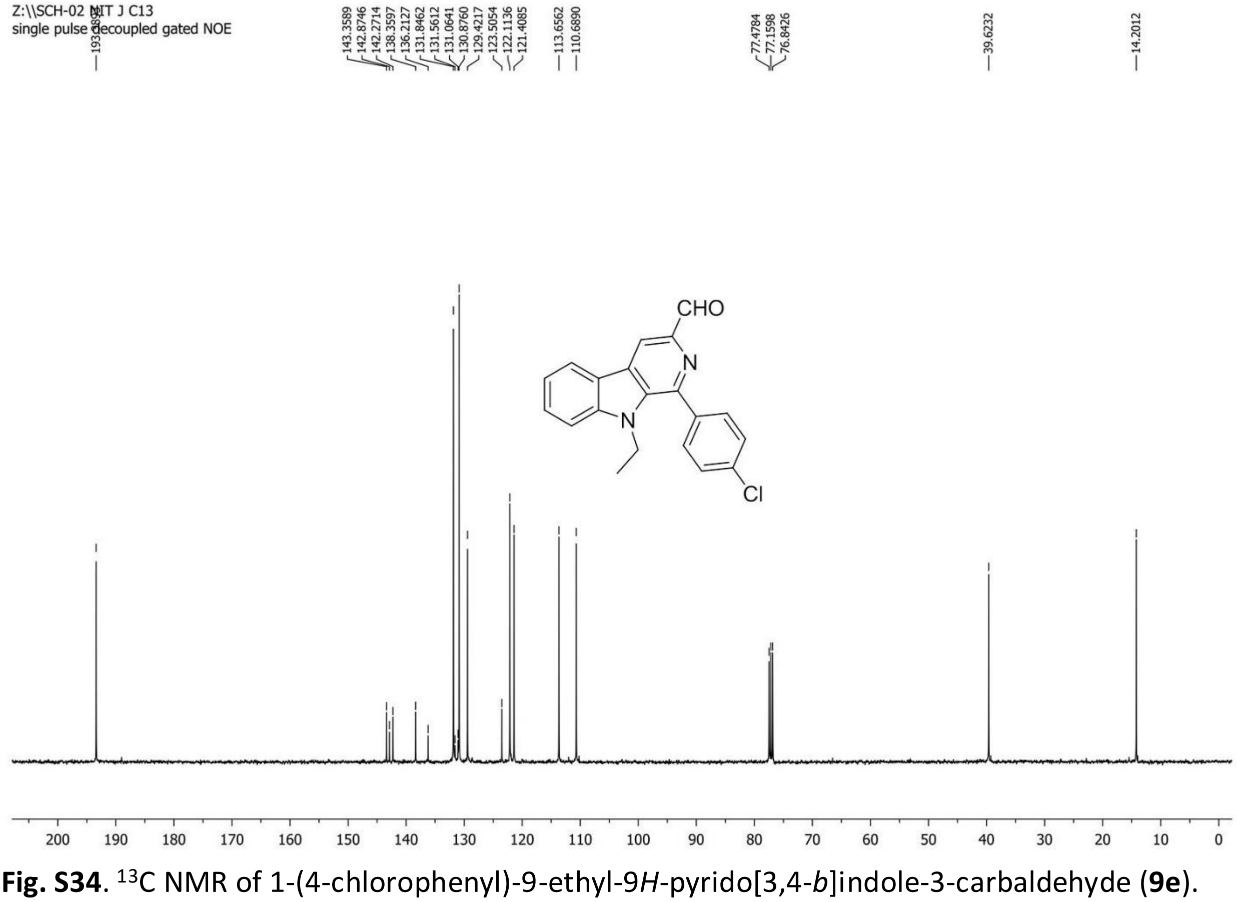

Fig. S34. ^{13}C NMR of 1-(4-chlorophenyl)-9-ethyl-9*H*-pyrido[3,4-*b*]indole-3-carbaldehyde (**9e**).

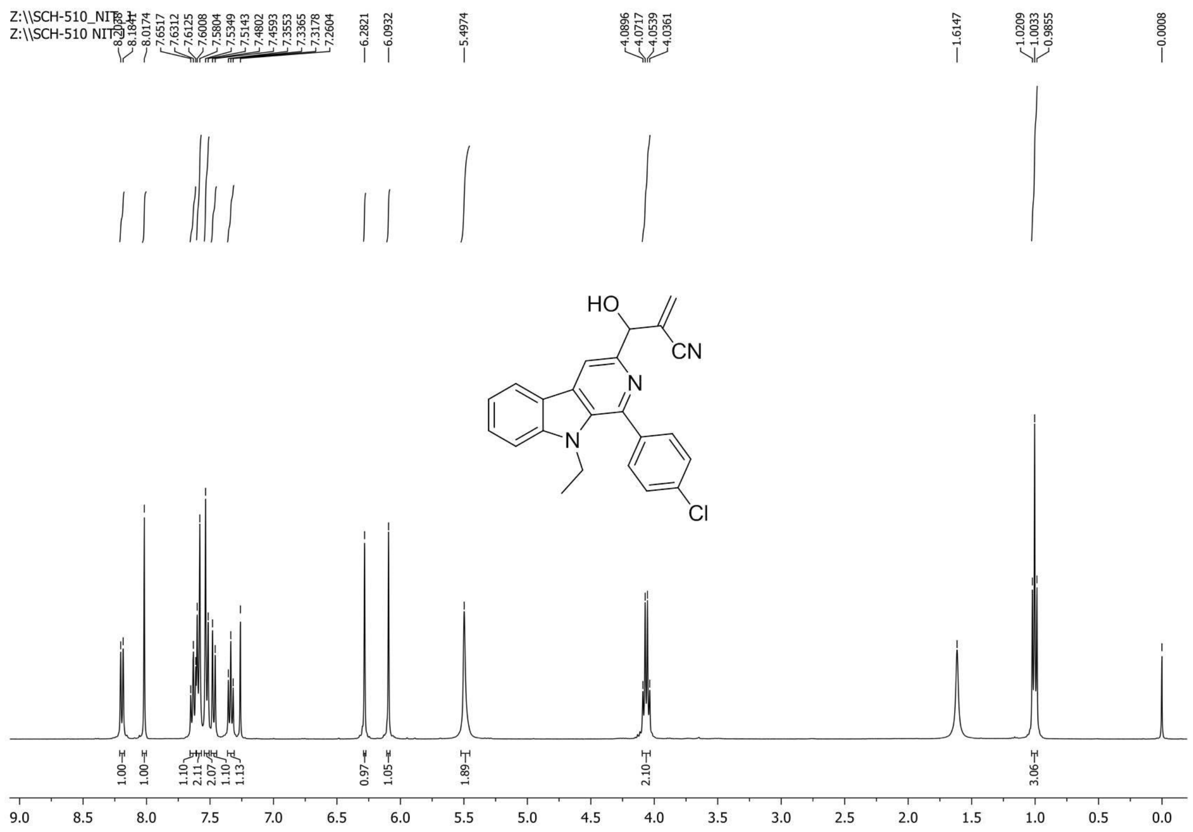

Fig. S35. ^1H NMR of 2-((1-(4-chlorophenyl)-9-ethyl-9*H*-pyrido[3,4-*b*]indol-3-yl)(hydroxy)methyl)acrylonitrile (**10eA**).

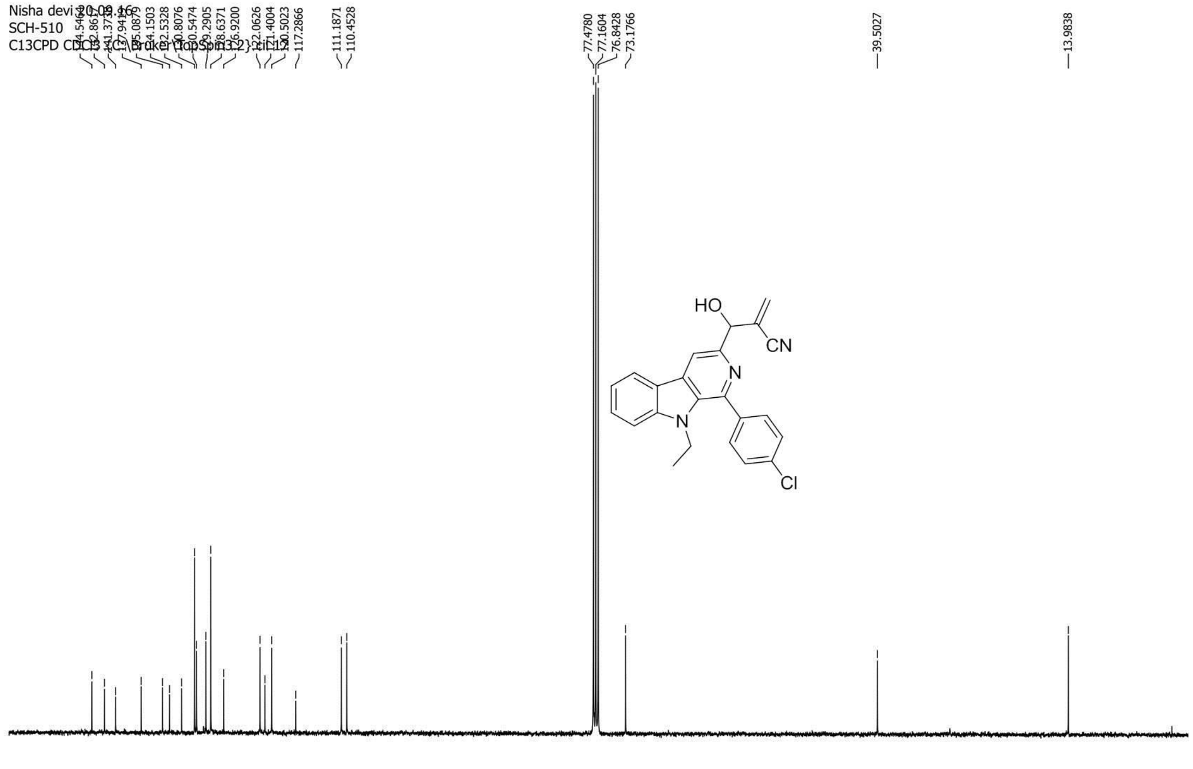

Fig. S36. ^{13}C NMR of 2-((1-(4-chlorophenyl)-9-ethyl-9*H*-pyrido[3,4-*b*]indol-3-yl)(hydroxy)methyl)acrylonitrile (**10eA**).

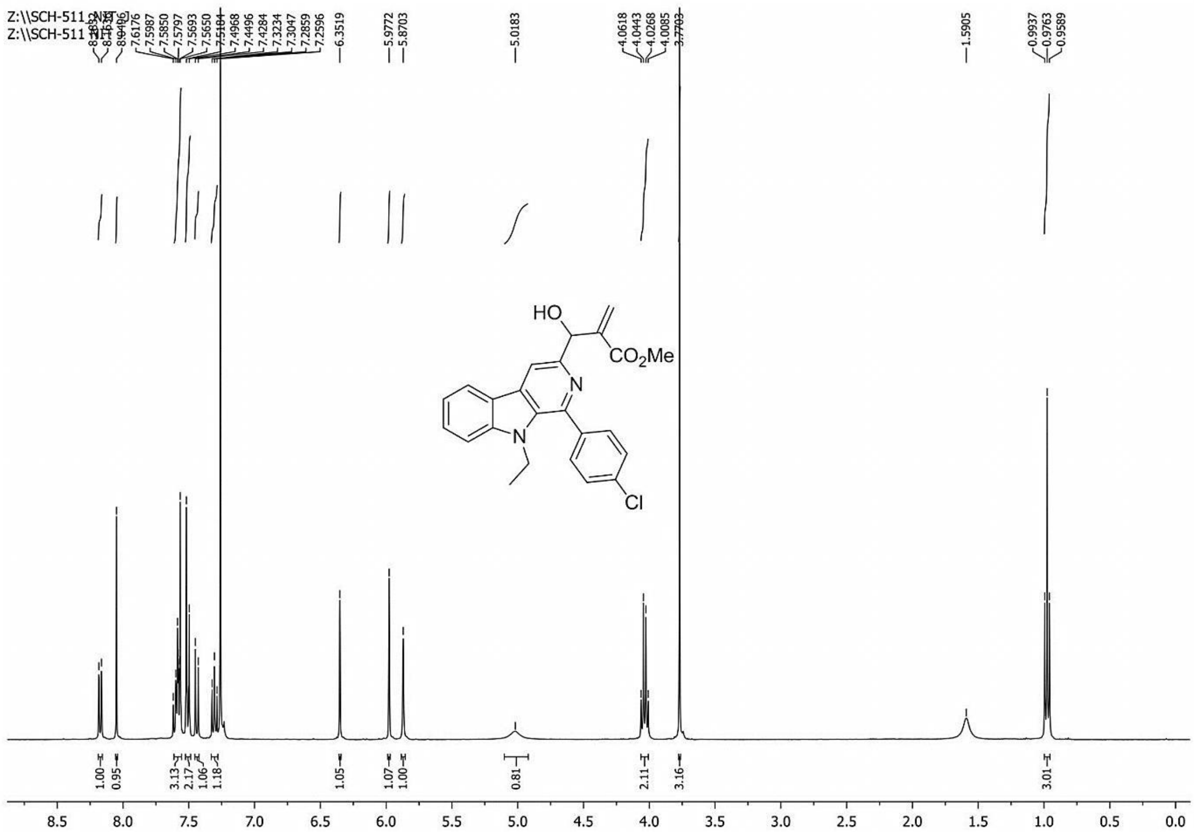

Fig. S37. ^{1}H NMR of methyl 2-((1-(4-chlorophenyl)-9-ethyl-9*H*-pyrido[3,4-*b*]indol-3-yl)(hydroxy)methyl)acrylate (**10eB**).

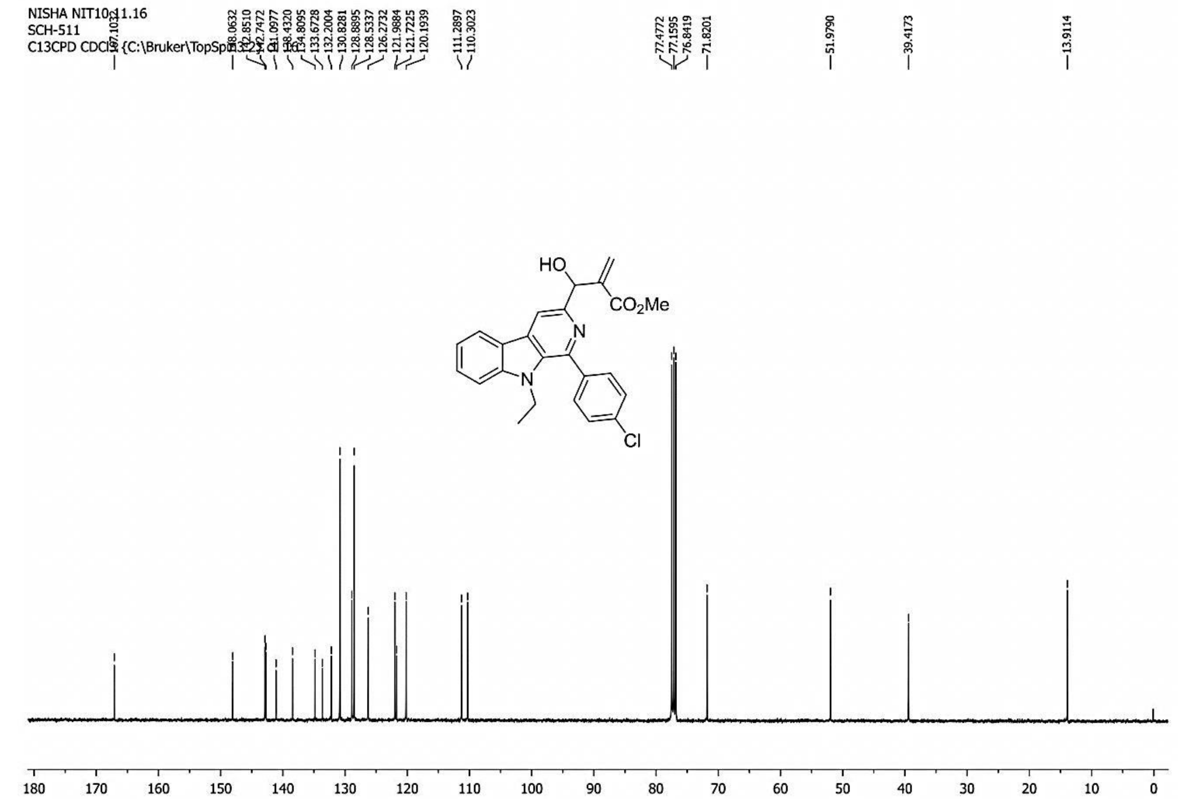

Fig. S38. ^{13}C NMR of methyl 2-((1-(4-chlorophenyl)-9-ethyl-9*H*-pyrido[3,4-*b*]indol-3-yl)(hydroxy)methyl)acrylate (**10eB**).

References

1. Singh, D.; Kumar, V.; Devi, N.; Malakar, C. C.; Shankar, R.; Singh, V. *Adv. Synth. Catal.* **2017,** *359,* 1213–1226. doi:10.1002/adsc.201600970
2. Devi, N.; Gupta, A.; Gujjarappa, R.; Malakar, C. C.; Singh, V. *Heterocycles* **2021,** *102,* 4, 705–722. doi:10.3987/COM-20-14403
